# Molecular Pathogenesis of Post-Transplant Acute Kidney Injury: Assessment of Whole-Genome mRNA and MiRNA Profiles

**DOI:** 10.1371/journal.pone.0104164

**Published:** 2014-08-05

**Authors:** Julia Wilflingseder, Judith Sunzenauer, Eva Toronyi, Andreas Heinzel, Alexander Kainz, Bernd Mayer, Paul Perco, Gábor Telkes, Robert M. Langer, Rainer Oberbauer

**Affiliations:** 1 Department of Nephrology and Dialysis, Medical University Vienna, Vienna, Austria; 2 Department of Nephrology and Transplantation, KH Elisabethinen, Linz, Austria; 3 Department of Transplantation and Surgery, Semmelweis University, Budapest, Hungary; 4 emergentec biodevelopment GmbH, Vienna, Austria; University of Torino, Italy

## Abstract

Acute kidney injury (AKI) affects roughly 25% of all recipients of deceased donor organs. The prevention of post-transplant AKI is still an unmet clinical need. We prospectively collected zero-hour, indication as well as protocol kidney biopsies from 166 allografts between 2011 and 2013. In this cohort eight cases with AKI and ten matched allografts without pathology serving as control group were identified with a follow-up biopsy within the first twelve days after engraftment. For this set the zero-hour and follow-up biopsies were subjected to genome wide microRNA and mRNA profiling and analysis, followed by validation in independent expression profiles of 42 AKI and 21 protocol biopsies for strictly controlling the false discovery rate. Follow-up biopsies of AKI allografts compared to time-matched protocol biopsies, further baseline adjustment for zero-hour biopsy expression level and validation in independent datasets, revealed a molecular AKI signature holding 20 mRNAs and two miRNAs (miR-182-5p and miR-21-3p). Next to several established biomarkers such as lipocalin-2 also novel candidates of interest were identified in the signature. In further experimental evaluation the elevated transcript expression level of the secretory leukocyte peptidase inhibitor (SLPI) in AKI allografts was confirmed in plasma and urine on the protein level (*p*<0.001 and *p* = 0.003, respectively). miR-182-5p was identified as a molecular regulator of post-transplant AKI, strongly correlated with global gene expression changes during AKI. In summary, we identified an AKI-specific molecular signature providing the ground for novel biomarkers and target candidates such as SLPI and miR-182-5p in addressing AKI.

## Introduction

Post-transplant acute kidney injury (AKI) is a frequent complication after kidney transplantation and is associated with shortened graft survival [1], [2], [3]. In spite of a remarkable progress in the field of transplantation medicine, the incidence of AKI over the last 20 years remained constant. Rates between 15 and 25% have been reported, which mainly depend on the definition of AKI but also showing substantial differences between centers [4], [5].

Several risk factors contributing to the development of AKI after kidney transplantation have been identified, including donor age, prolonged cold and warm ischemic times, and the use of organs procured from non-heart beating donors [5], [6]. Molecular mechanisms determining the fate of kidney allografts via triggering the development of AKI remain unclear as ischemia and reperfusion injury (IRI) occur at a varying degree in all transplanted organs.

Molecular and cellular alterations associated with IRI mainly include tissue damage caused by reactive oxygen species (ROS), nitric oxide or peroxynitrite [7], but also a decreased adenylate cyclase activity and consecutive low intracellular cAMP levels. Consequences are impaired endothelial cell barrier functions (vascular permeability and leakage) [8], [9]. Furthermore, significant alterations at the transcriptional level in control of gene expression occur, such as upregulation of the hypoxia inducible factor 1A (HIF1A) or the nuclear factor kappa B (NF-kB), the central regulator of inflammatory response [10]. Later on in the response cascade of IRI, cell death via apoptosis and necrosis is triggered [11]. Additionally, the activation of innate and adaptive immune response is provoked and contributes to tissue damage and injury, comprising pattern recognition receptors (PRRs) such as Toll-like receptors (TLRs) and/or infiltration of macrophages and/or T-cells [12].

Furthermore, microRNAs (miRNAs) were identified to be involved in the molecular cascade following IRI. miRNAs are post-transcriptional modulators that regulate target genes by perfect- or semi-complementary base pairing, finally resulting in RNA degradation or inhibition of translation, respectively [13]. Godwin et al. and Shapiro et al. reported miRNA signatures after warm ischemia followed by reperfusion in mice kidneys [14], [15], and our group recently showed a deregulation of miRNAs in human post-transplantation (TX) AKI biopsies [16]. However, our knowledge on how miRNAs regulate gene expression in injured kidney tissue and impact transcript and protein levels is fairly understood.

This study was designed to specifically elucidate molecular regulation patterns on the combined level of mRNA and microRNA expression of transplant kidneys diagnosed with AKI versus grafts showing prompt organ function.

## Materials and Methods

### Renal transplant biopsy specimen

The study protocol was approved by the Institutional Review Board (IRB) (Ethical Committee of the Semmelweis University Budapest # 20303-0/2010-1018EKU (821/PI/01)), and all recipients provided written informed consent to follow-up allograft biopsy and blood and urine samples for research. There was no need for informed consent for zero-hour biopsies from deceased donor organs according to the IRB protocol, as allowance to perform autopsy existed. Living donors gave written informed consent to zero-hour donor biopsy for research.

We prospectively collected zero-hour biopsies from 166 renal allograft recipients from March 2011 to April 2013. From these 166 recipients, 34 had a follow-up kidney biopsy within 12 days after transplantation with the following clinical conditions: (i) 16 rejections (eight Banff 1, two Banff 2, one antibody-mediated rejection, two Banff 1 rejections with humoral rejection signs and three Banff Borderline rejections), (ii) eight with acute tubular necrosis without rejection defined as AKI and (iii) ten protocol biopsies without pathology acting as control group (primary graft function). AKI was defined as acute tubular necrosis in the absence of cellular and/or humoral signs of rejection according to the Banff 2012 criteria [17]. The indication for AKI kidney biopsy was more than one dialysis session within the first week after transplantation and a serum creatinine above 4 mg/dL after the first week. Together with the post-TX biopsies, EDTA-plasma and urine was collected from the recipients. The biopsy specimens were immediately submerged in RNAlater™ (Ambion, Austin, Texas, USA) ensuring maximal RNA quality. For the present study we used (i) eight biopsy pairs with histological lesions of acute tubular necrosis in the post-TX biopsies (AKI group) and (ii) ten allografts with primary function and protocol biopsies without pathology (PGF group).

### RNA Extraction and Microarray Hybridization

Total RNA was isolated and purified using chloroform and trizol reagent (Invitrogen, Carlsbad, California USA). RNA yield and quality were checked with the Agilent 2100 Bioanalyzer (Agilent, Palo Alto, California, USA). Kidney mRNA expression analysis was performed according to the NuGEN-recommended protocol using the Affymetrix GeneChip Human Gene 2.0 ST Array. Affymetrix GeneChip miRNA 3.0 Arrays were used for miRNA profiling. Total RNA (1 µg per sample) was labeled using the FlashTag™ Biotin HSR RNA Labeling Kit (Affymetrix, Santa Clara, California, USA) and hybridized to the arrays as described by the manufacturer.

### miRNA and mRNA microarray analysis

Affymetrix data were pre-processed, normalized, and summarized using the robust multi-average (RMA) method with quantile normalization, and annotated using the corresponding annotation file (.cdf) in Bioconductor. Microarray raw data files are available in the Gene Expression Omnibus (GEO) at NCBI with the accession number GSE53773. Interquartile range and intensity filtering were used to exclude features with low variance and signal intensity (log_2_ level <4) over all profiles [18]. Significance Analysis of Microarrays (SAM) was used to determine differentially regulated mRNAs and miRNAs comparing AKI and control group samples [19]. The false discovery rate (FDR) was set to <10% and the minimum fold change to 1.5 in order to detect significantly differentially regulated transcripts. Further, the derived mRNA and miRNA AKI signatures were verified in independent datasets [16], [20]. Principal component analysis was performed based on the covariance matrix to visualize the variance in the gene expression signatures of the allografts. Spearman correlation was applied to evaluate association of differentially regulated miRNAs and mRNAs. Functional grouping of genes was based on Gene Ontology (GO) terms utilizing the DAVID Functional Annotation platform [21]. Three algorithms were used to predict miRNA targets, namely DIANAmT, miRanda and Targetscan [22], [23], [24]. Only miRNA targets identified by all three prediction algorithms were included in further analysis. Additionally, experimentally validated targets were extracted for identified miRNAs from miRTarBase [25].

To extract biomarker candidates discussed in the context of AKI the text mining tool FABLE (http://fable.chop.edu/overview.jsp) was used [26], taking into account all genes having at least two scientific references with the following search terms: ‘Acute kidney injury’ and ‘Biomarker’.

### Quantitation of SLPI protein concentration

We used a quantitative sandwich enzyme-linked immunoassay (ELISA) to determine the protein concentration of SLPI in plasma and urine as described by the manufacturer (R&D Systems, Minneapolis, MN, USA, article# DPI00).

### Statistical analysis of clinical data

Continuous data were analyzed by non-parametric Wilcoxon Rank Sum Test. Categorical data were evaluated by Chi-square test or Fisher's exact test where appropriate. A *p*-value below 0.05 was considered statistically significant. Statistical assessment was performed with SAS for Windows 9.3 (The SAS Institute, Inc., Cary, North Carolina, USA).

## Results

### Patient characteristics

We first analyzed two sequential biopsies in 18 different kidney grafts (Basic mRNA and miRNA dataset). The first biopsy was carried out at implantation after cold storage (zero-hour biopsy) and the second biopsy was taken within 12 days after transplantation. Eight allografts fulfilled histological criteria for acute tubular injury in the post-TX biopsy (AKI group), and the other ten allografts had primary graft function (PGF) serving as control group with no pathology in the post-TX biopsy (protocol biopsy). Demographic data on the 18 transplant donors and recipients are provided in Table 1. None of the demographic variables of the recipient and donor pairs were significantly different between the two groups. Median donor age was numerically higher in the AKI group (53 years; interquartile range (IQR): [46.8; 56.8]) when compared to the PGF group (42.3 years; IQR: [40.3; 50], *p* = 0.28). All deceased donors received catecholamines before organ retrieval. Median serum creatinine level at time of post-TX biopsy was 9.81 mg/dL in the AKI group and 1.54 mg/dL in the PGF group (*p*<0.001). Independent publicly available mRNA and miRNA datasets were used for validation of findings in the Basic mRNA and miRNA dataset. The independent mRNA dataset comprised renal post-TX biopsies from allografts diagnosed with AKI (n = 28) and six week protocol biopsies (n = 11) published by Famulski et al. in 2012 [20] and the independent miRNA dataset consisted of miRNA profiles of AKI post-TX biopsies (n = 14) and three-month protocol biopsies (n = 10) from one of our previous studies [16]. Patient characteristics from these published datasets were extracted from the original publications and are also provided in Table 1. A significant difference was found in biopsy collection time between the AKI and protocol biopsy groups and no zero-hour biopsies were collected in these studies. Variables not found in the original publications are indicated as ‘not stated’.

**Table 1:** Kidney donor and recipient characteristics comparing AKI and control groups in the Basic and independent validation datasets.

	*Basic mRNA and miRNA dataset*			*Independent mRNA dataset Famulski et al. * [*20*]			*Independent miRNA dataset Wilflingseder et al. * [*16*]		
Variable	PGF	AKI	*p*-value	Protocol Biopsy Cohort	AKI	*p*-value	Protocol Biopsy Cohort	AKI	*p*-value
Number of total biopsies	20	16	na	11	28	na	10	14	na
Number of 0 h biopsies	10	8	na	0	0	na	0	0	na
Number of donor organs	10	8	na	10	26	na	10	13	na
Donor age (years)	42.5 (40.3; 50)	53 (46.8; 56.5)	0.284	55 (20–82)^b^	53 (22–69)^b^	>0.05	59.5 (54.0; 67.0)	51.0 (46.0; 64.0)	0.42
Donor sex (m/f)	4/6	5/3	0.637^a^	n. st.	n. st.	n. st.	6/4	5/8	0.41
Donor source (living/deceased)	0/10	2/6	0.183^a^	2/9	11/15	>0.05	6/4	0/13	<0.01^a^
Donor last creatinine (mg/dl)	0.94 (0.85; 1.19)	0.92 (0.78; 1.06)	0.475	n. st.	n. st.	n. st.	0.82 (0.69; 0.92)	0.95 (0.7; 1.4)	0.17
Recipient sex (m/f)	8/2	6/2	1.000^a^	n. st.	n. st.	n. st.	3/7	2/11	0.62
Recipient age (years)	57.6 (47.9; 62.9)	55.3 (50.1; 58.7)	0.824	49 (29–70)^c^	52 (16–75)^c^	>0.05	50.8 (41.3; 59.1)	53.5 (47.2; 65.1)	0.34
Transplant number (1/2/3/4/5)	9/1/0/0/0	6/0/2/0/0	0.183^a^	n. st.	n. st.	n. st.	10/0/0/0/0	10/1/1/0/1	1.00^a^
Cold ischemic time (hours)	13.5 (11.9; 17.6)	12.8 (7.5; 15.5)	0.398	n. st.	n. st.	n. st.	1.0 (0.0; 8)	11.5 (10; 17.5)	<0.01
PRA latest (%)	0 (0; 4.5)	2 (0; 18)	0.363	n. st.	n. st.	n. st.	0.0 (0.0; 0.0)	0.0 (0.0; 5.0)	0.02
Sum of HLA mismatches (0/1/2/3/4/5)	2/0/2/4/1/1	0/1/0/2/1/4	0.231^a^	n. st.	n. st.	n. st.	0/1/2/4/2/1	3/3/1/4/1/1	0.57
Immunosuppression (CNI/else)	9/1	8/0	1.000^a^	n. st.	n. st.	n. st.	9/1	12/1	1.00^a^
Induction therapy (none/IL2/ATG)	8/1/1	6/1/1	1.000^a^	n. st.	n. st.	n. st.	6/3/1	11/1/1	0.26^a^
Time from TX to biopsy (days)	7.5 (6.3; 9.8)	7 (6; 9.5)	0.651	42	16 (6–42)^c^	<0.001	104.5 (102; 109.5)	7 (2; 8)	<0.001
Serum creatinine at post-TX biopsy	1.54 (1.32; 1.73)	9.81 (5.74; 10.14)	<0.001	n. st.	n. st.	n. st.	n. st.	n. st.	n. st.

Continuous data are provided as median and 1^st^ and 3^rd^ quartile; categorical data are shown as counts.

na … not applicable,

a Fisher's exact test,

b mean (range),

c median (range),

n. st. … not stated.

PGF … primary graft function, AKI … acute kidney injury.

### Molecular phenotype of post-transplant AKI

#### Differentially regulated genes in the injured kidney

In a first analysis we compared mRNA profiles of post-TX biopsies between the AKI (n = 8) and PGF group (n = 10) using the SAM method setting the FDR to <10%. In total, 245 differentially regulated genes were identified showing fold changes >1.5 (Table S1). Based on the 245 differentially regulated genes, principal component analysis (PCA) was performed. Samples were clustered utilizing the first three principal components that captured over 85% of the variance in the dataset (Figure S1).The 18 allografts separated into two distinct clusters in line with pathology stages, indicating that AKI allografts could be characterized based on the 245 differentially regulated genes. Functional annotation of the 245 genes revealed several significantly over-represented biological processes such as ‘response to wounding’, ‘response to toxin’, or ‘response to metal ion and oxidation-reduction’ (Table S2).

In a second step we also factored the mRNA expression levels of the zero-hour biopsies into the analysis by calculating the log_2_ expression difference between the two sequential biopsies of each allograft, and then again comparing AKI allografts vs. allografts with primary graft function (baseline adjustment). This analysis allowed us to identify the expression level trajectory in the development of AKI from implantation to the histological diagnosis of AKI in the recipient. 39 genes of the initial set of 245 genes still showed significant deregulation on the transcript level in the development of AKI (Table S3).

To assess the validity of the identified differentially regulated genes we evaluated our results in the mRNA dataset from Famulski et al., 2012 (GEO accession number: GSE30718) [20]. After re-analysis of this mRNA expression dataset utilizing the same statistical workflow as applied for our profiles, 932 genes were identified as significantly differentially regulated between the 28 AKI samples and 11 protocol biopsies without signs of AKI. A significant overlap was observed for our set of 39 baseline-adjusted AKI genes, with 20 also being present in the dataset from Famulski et al. (Chi-square test, *p*<0.001, using the total number of shared features on the Affymetrix arrays as reference, Table 2). The 20 shared genes could be categorized into the following biological processes: (i) ‘response to wounding’ (LYVE1, S100A8, SERPINA3, CD163; *p* = 0.02), (ii) ‘acute-phase response’ (SERPINA3, CD163; *p* = 0.04) and (iii) ‘inflammatory response’ (S100A8, SERPINA3, CD163; *p* = 0.04).

**Table 2:** AKI mRNA signature as verified in the independent evaluation dataset.

		post-TX		baseline adjusted		
Probe Set ID	Gene Symbol	raw p-value	Fold change	raw p-value	Fold change	Gene Description
16919547	SLPI	1.55E-03	5.67	1.20E-03	15.17	secretory leukocyte peptidase inhibitor
16775083	OLFM4	6.15E-03	2.86	2.98E-03	7.88	olfactomedin 4
16787902	SERPINA3	1.08E-03	2.03	2.91E-03	7.03	serpin peptidase inhibitor, clade A (alpha-1 antiproteinase, antitrypsin), member 3
**16743647**	**MMP7**	**3.42E-02**	**2.88**	**1.54E-02**	**4.06**	**matrix metallopeptidase 7 (matrilysin, uterine)**
16760792	CD163	2.77E-02	1.94	1.69E-02	3.12	CD163 molecule
**16693414**	**S100A8**	**7.43E-04**	**2.40**	**1.66E-02**	**3.09**	**S100 calcium binding protein A8**
16735751	LYVE1	7.55E-04	1.53	2.09E-03	2.28	lymphatic vessel endothelial hyaluronan receptor 1
**17089525**	**LCN2**	**5.02E-03**	**1.95**	**8.81E-03**	**2.28**	**lipocalin 2**
16707503	EXOC6	8.75E-04	1.52	1.88E-03	1.78	exocyst complex component 6
17024144	IFNGR1	4.61E-03	1.51	7.64E-03	1.77	interferon gamma receptor 1
17087615	LPPR1	5.54E-04	0.51	3.00E-03	0.57	lipid phosphate phosphatase-related protein type 1
16991527	CYFIP2	2.00E-03	0.55	2.80E-03	0.52	cytoplasmic FMR1 interacting protein 2
16695262	KCNJ10	3.92E-04	0.55	3.00E-04	0.52	potassium inwardly-rectifying channel, subfamily J, member 10
17101262	ARSF	1.46E-03	0.57	3.30E-03	0.47	arylsulfatase F
17094946	TRPM6	1.89E-02	0.62	2.19E-03	0.45	transient receptor potential cation channel, subfamily M, member 6
17072059	SLC30A8	2.29E-02	0.53	4.78E-03	0.45	solute carrier family 30 (zinc transporter), member 8
16773086	FGF9	4.37E-04	0.55	8.50E-04	0.45	fibroblast growth factor 9 (glia-activating factor)
16934643	PVALB	2.02E-04	0.39	2.75E-03	0.42	parvalbumin
17007950	PNPLA1	1.15E-03	0.50	1.10E-03	0.36	patatin-like phospholipase domain containing 1
16962671	TMEM207	1.57E-04	0.32	9.31E-04	0.33	transmembrane protein 207

**bold**......molecular features discussed as biomarker candidates of acute kidney injury.

Raw p-values and fold changes of verified differentially regulated genes are provided.

Several of these deregulated genes were shown to be AKI biomarker candidates already discussed in the scientific literature according to FABLE analysis (highlighted bold in the gene lists, Table S1, S3 and Table 2), including MMP7, LCN2 and S100A8, supporting the validity of the identified AKI signature (Table 2).

#### Differentially regulated miRNAs in AKI

Deregulated miRNAs were identified following the same analysis workflow as used for deriving the mRNA profiles. 49 microRNAs were significantly upregulated in the AKI post-TX biopsies by more than 1.5 fold (SAM, FDR <10%, Table S4) compared to the PGF group. miRNAs with significant down-regulation were not detected. After expression baseline adjustment taking expression levels of the zero-hour biopsies into account, 29 miRNAs remained significantly differentially regulated (Table S5). For verification of the identified miRNAs a dataset recently published by our group was used (GEO accession number: GSE30282) [16]. We again re-analyzed this dataset by comparing miRNA profiles of AKI post-TX biopsies (n = 14) and three-month protocol biopsies (n = 10) of kidneys with no AKI history, and identified twelve miRNAs as significantly upregulated. Of these 12 miRNAs from our validation set, two miRNAs (miR-21-3p and miR-182-5p) were found in the baseline-adjusted list of 29 miRNAs (Table 3). Only five out of the 29 differentially regulated miRNAs were present as probes on the Affymetrix array that was used in our validation dataset. Therefore an overlap of two miRNAs was still statistically significant when taking into account the number of common and valid probes of mature miRNAs on the two different arrays (Fisher's exact test, p = 0.03).

**Table 3:** AKI miRNA signature as verified in the evaluation dataset.

			post-TX		baseline adjusted	
Probe Set ID	miRNA Name	miRBase Accession	raw *p*-value	Fold change	raw *p*-value	Fold change
hsa-miR-21-star_st	hsa-miR-21-3p	MIMAT0004494	1.64E-02	2.22	2.41E-02	3.34
hsa-miR-182_st	hsa-miR-182-5p	MIMAT0000259	1.62E-02	1.56	2.46E-03	1.88

Raw p-values and fold changes of verified differentially regulated miRNAs are provided.

**Figure 1:**
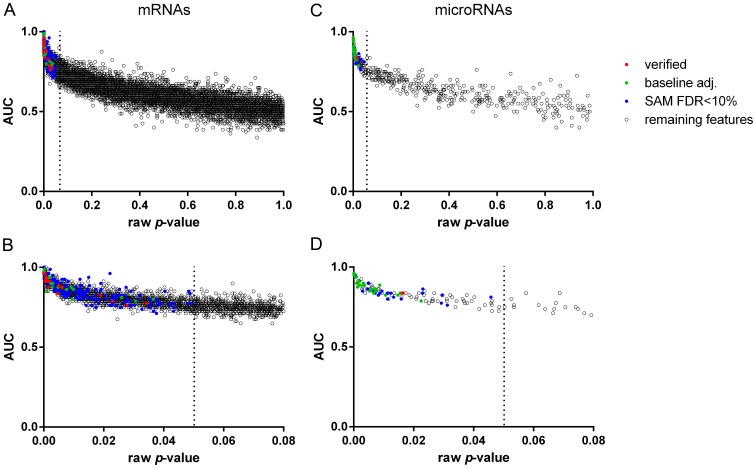
Raw *p*-values and area under the curve (AUC) of the investigated mRNAs and miRNAs comparing post-TX AKI and PGF allografts. (A) full p-value range of mRNAs (B) mRNAs with *p*-value<0.08 on the x-axis for specific visualization of features with a raw *p*-value<0.05 (C) full p-value range of microRNAs (D) microRNAs with raw *p*-value<0.08 on the x-axis; red: features verified in an independent dataset (20 mRNAs and two microRNAs), green: significant features after baseline adjustment (39 mRNAs and 29 miRNAs), blue: significant features identified by SAM (245 mRNAs and 49 microRNAs), black: remaining features (11,788 mRNAs and 393 microRNAs).

Figure 1 shows the raw *p*-values of the investigated mRNAs and miRNAs resulting from the group comparisons of post-TX AKI and PGF allografts plotted against the area under the curve (AUC) of the receiver operating characteristic (ROC) curve computed for each individual transcript. Significantly differentially regulated mRNAs and miRNAs are highlighted in color. Indeed, baseline adjustment and verification in an independent dataset led to selection of features with a lower *p*-value and a higher AUC value, strengthening the utility of our analysis approach.

### MicroRNA-182-5p expression is correlated with global gene expression response of post-transplant AKI

**Figure 2:**
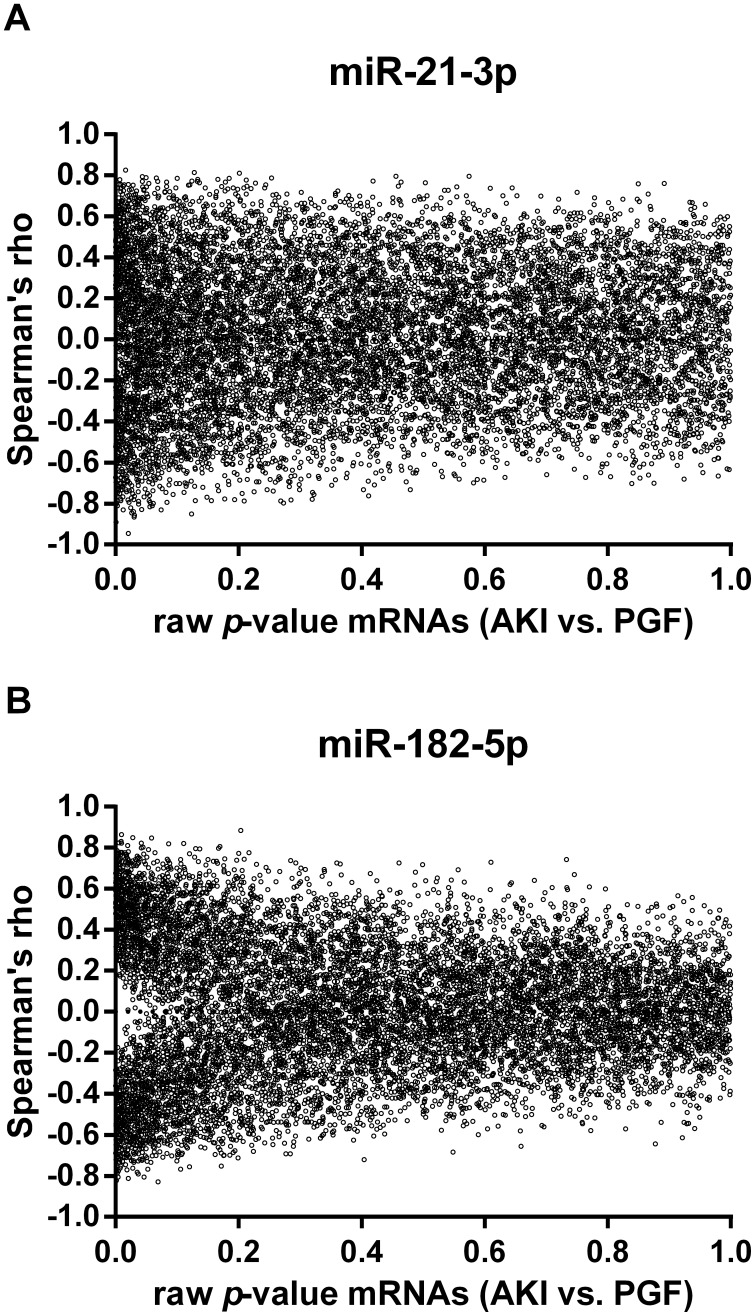
Correlation coefficients (Spearman's rho) of (A) miR-21-3p and (B) miR-182-5p and mRNAs plotted against the corresponding raw *p*-values of baseline adjusted mRNA levels comparing the AKI and PGF group.

Spearman correlation was used to associate miRNA and mRNA expression levels. Correlation coefficients of the verified miRNAs, miR-21-3p and miR-182-5p, were calculated for the baseline-adjusted mRNA expression levels and plotted against the raw *p*-values of the mRNAs comparing AKI and PGF allografts (Figure 2). Apparently, the expression levels of miR-182-5p showed a more pronounced correlation with the differentially regulated mRNAs as indicated by the funnel at lower p-values in Figure 2B. Correlation with miR-21-3p expression levels did not exhibit this effect (Figure 2A) suggesting a central role of miR-182-5p in graft injury.

We identified three further miRNAs (miR-132-3p, miR-212-3p and miR-149-3p) not being covered in the verification data set with a similar correlation pattern as seen for miR-182-5p (Figure S2). Together these four miRNAs are highly correlated (*R*<−0.7 or >0.7) to 26 baseline adjusted differentially regulated mRNAs (Table S6), which can be categorized into the biological processes ‘ECM-receptor interaction’ (*p* = 0.004) and ‘Focal adhesion’ (*p* = 0.02). Further two genes with a negative correlation coefficient are potential direct targets of two miRNAs based on three miRNA target prediction algorithms (DIANAmT, miRanda and TargetScan). miR-182-5p is predicted to be a potential inhibitor of klotho (KL) and miR-149-3p of potassium inwardly-rectifying channel, subfamily J, member 10 (KCNJ10). None of the correlated genes were experimentally verified targets of the four miRNAs according to miRTarBase.

### Validation of a potential novel biomarker of AKI in plasma and urine

**Figure 3:**
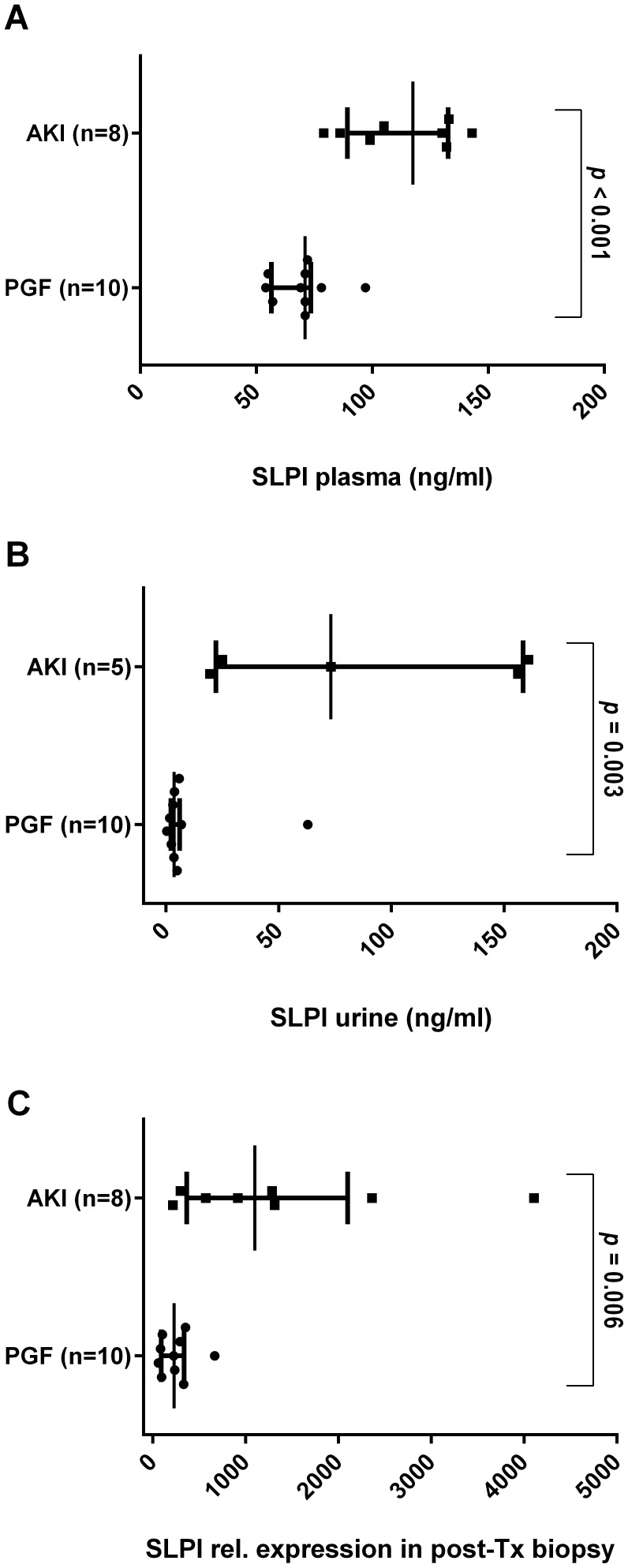
SLPI protein concentration (ng/ml) measured by sandwich ELISA in (A) EDTA-plasma and (B) urine of AKI and PGF patients. Three AKI patients were anuric at the time of post-TX biopsy. (C) Relative gene expression levels of SLPI in post-TX biopsies. Individual data points as well as median, 1^st^ and 3^rd^ quartile are provided.

To further assess the validity of our findings also on the protein level, we selected the protein product of the gene revealing the strongest association with AKI according to mRNA expression fold change, namely the secretory leukocyte peptidase inhibitor (SLPI). SLPI protein concentrations were determined in EDTA-plasma and urine of the 18 kidney allograft recipients. EDTA plasma and urine were collected at the same day as the post-TX biopsy. For three AKI patients only plasma was available due to lack of urine excretion. SLPI protein concentration was found to be significantly elevated in plasma as well as in urine of AKI patients when compared to the PGF group (Figure 3). Median SLPI concentration in plasma was 117 ng/ml (IQR: [96; 132]) in the AKI group and 71 ng/ml (IQR: [60; 72]) in the PGF group (*p*<0.001, Figure 3A). In urine the SLPI concentration was 73 ng/ml (IQR: [25; 156]) in the AKI group (n = 5) and 3.6 ng/ml (IQR: [2.5; 5.6]) in the PGF group (*p* = 0.003, Figure 3B). SLPI gene expression levels in the transplant kidneys were also highly correlated with SLPI protein concentrations in urine (*R* = 0.77) and plasma (*R* = 0.57), respectively. Relative gene expression of SLPI in post-TX biopsies is shown in Figure 3C.

## Discussion

This study provides a comprehensive molecular picture of post-transplant AKI on the combined mRNA and miRNA level. As all transplant organs experienced IRI, the comparison between AKI and time-matched biopsies from allografts with primary function within the first days after transplantation allowed us to investigate the molecular profiles leading to poor initial graft function and acute tubular injury. Several known as well as novel molecules were identified.

The clinical consequences of post-transplant AKI leading to delayed graft function (DGF) were extensively investigated. A meta-analysis of 33 studies showed that recipients with DGF had an increase of relative risk (RR) by 41% to lose their graft in a median follow-up time of 3.2 years [2]. Further, DGF was associated with an augmented rate of acute rejection episodes (RR 18%–66%) and higher serum creatinine levels at the end of the follow-up period [2], [27], [28]. Therefore, prevention of DGF is of significant medical relevance, especially regarding limited donor pools and the acceptance of further marginal donor kidneys [29], [30], [31], [32].

Our current understanding of the molecular mechanisms underlying post-transplant AKI is mainly based on investigations of IRI [33] and of zero-hour kidney transplant biopsies [34], [35], [36]. To our knowledge only one study so far analyzed the transcriptome of human renal allograft biopsies during AKI [20] which was also used as independent validation set for findings of the current study. Six-week protocol biopsies of allografts with no history of AKI and stable kidney function (n = 11) served as the control group in this study. The validation study for our miRNA findings was based on protocol biopsies procured after three months [16]. Aside these differences in follow-up time a significant overlap of deregulated mRNAs and miRNAs was found, providing us with a stable molecular AKI signature on mRNA and miRNA level. Several biomarker candidates were found in the list of differentially regulated genes, with some of them discussed as prognostic markers regarding future graft function such as lipocalin-2 (LCN2) and Kidney Injury Molecule 1 (HAVCR1) [37], [38], [39], allowing to deduce an association with intrinsic injury in the kidney linked to outcome. Hence, the consensus mRNA and miRNA AKI signature may be utilized to establish biomarkers providing a better approximation of the injury status in the organ. We evaluated one of these candidates, SLPI, on the protein level in plasma and urine of the transplant recipients. We could confirm the significantly elevated SLPI mRNA expression in injured allografts both in plasma and urine of AKI patients also on the protein level. Furthermore SLPI urine concentration was highly correlated with SLPI gene expression in the organ, indicating a link of elevated SLPI concentration in urine to the status of kidney damage in the organ itself. Tubule epithelial cells have already been identified by immunohistochemistry as the source of SLPI protein expression in the kidney [40]. However, further evaluation in larger patient cohorts is needed to assess the applicability of SLPI as a novel biomarker for AKI.

On the molecular level the most challenging part is to distinguish repair response from mechanisms causing devastating injury in the donor organ and subsequent detrimental effects on graft and patient survival. Final proof can only be accomplished in functional studies of the involved molecules. However, unbiased explorative analyses such as the present study are important for discovery, hypothesis generation and refinement. Therapeutic targets currently under investigation for DGF and IRI are summarized in two recently published reviews [5], [41]. Inhibition of the Toll-like receptor 2 (TLR2) is one of the experimental strategies (clinicaltrials.gov: NCT01794663). TLR signaling leads to an accelerated immune response and can be activated by so called ‘danger signals’ (i.e. from damaged tissue) [42]. We found a higher expression of TLR2 in AKI allografts (Table S1).

miRNAs are currently under intense investigation as therapeutic agents and potent modifiers of immune response and tolerance [43]. miRNAs are shown to regulate entire molecular networks via simultaneous targeting of several hundred genes [44]. This makes miRNAs, in contrast to compounds targeting a single molecule, interesting for clinical phenotypes such as AKI where entire cellular processes are deregulated.

miR-182-5p expression is profoundly correlated with genes identified to be strongly associated with kidney tissue injury. miR-182-5p can be activated by IL2 and STAT5 and inhibits FOXO1 expression [45]. FOXO1 acts as a master cellular regulator of a variety of cellular processes including cell survival, apoptosis, proliferation and metabolism, and also plays a critical role in the homeostasis of cells of the immune system including T-cells, B-cells and neutrophils [46]. Further, the absence of FOXO1 was shown to severely curtail the development of FOXP3+ regulatory T-cells (Tregs). Those Tregs that nevertheless developed were found to be non-functional *in vivo* and down-regulation of FOXO1 in T-cells resulted in lymphocyte infiltration [47]. Further, BCL2 is a direct target of miR-182-5p and inhibition of miR-182-5p resulted in a higher protein expression of BCL2, suggesting potent anti-apoptotic effects [48], [49].

Certainly there are limitations in the interpretation of the given kidney biopsy mRNA and miRNA profiles such as the limited sample size, which we aimed to compensate by controlling the FDR, and importantly via validation in independent sample cohorts. Additionally we confirmed differentially regulation of three mRNAs in the Basic dataset and two miRNAs in the independent miRNA dataset by qRT-PCR (Figure S3). Further, as microRNAs are post-transcriptional regulators which mainly inhibit translation of mRNAs into proteins [44], we based the identification of miRNA regulators on correlation analysis with the corresponding mRNA profiles of each allograft. This allowed us to capture the global impact on gene expression of the microRNA regulators [50].

Taken together, an AKI-specific molecular signature on the mRNA and miRNA level was identified. The identified molecules and processes set the stage for subsequent injury specific biomarker validation and functional studies uncovering the relation to immune-associated damage and repair response. This will lead to further improvement in understanding and potential clinical management of AKI.

## Supporting Information

Figure S1 **Principal component analysis based on the 245 differentially regulated genes comparing post-TX AKI and PGF allografts.** The first three principal components (PC) were plotted, capturing 85% of the variance in the dataset. Acute kidney injury allografts (grey spheres) and allografts with primary kidney function (black spheres) form two distinct clusters.
(DOCX)

Figure S2 **Correlation coefficients (Spearman's rho) of (A) miR-132-3p and (B) miR-212-3p (C) miR-149-3p calculated for all mRNAs against the raw p-values of baseline adjusted mRNA levels between the AKI and PGF group.**
(DOCX)

Figure S3 **qRT-PCR validation of significantly differentially regulated (A) mRNAs (SLPI, MMP7 and LCN2) and (B) miRNAs (miR-182-5p and miR-21-3p) between AKI and control group (PGF).** Log_2_ (relative expression) values are shown for the qRT-PCR and the array experiment. Individual data points as well as median, 1^st^ and 3^rd^ quartile are provided.
(DOCX)

Table S1 **245 significantly differentially regulated mRNAs comparing post-TX AKI and protocol biopsies from allografts with primary graft function.**
(DOCX)

Table S2 **Significantly over-represented biological processes embedded in the post-TX mRNA signature (245 mRNAs).**
(DOCX)

Table S3 **39 significantly differentially regulated mRNAs comparing AKI and PGF allografts after baseline adjustment.**
(DOCX)

Table S4 **Significantly differentially regulated miRNAs comparing post-TX AKI and protocol biopsies from allografts with primary graft function.**
(DOCX)

Table S5 **Significantly differentially regulated microRNAs comparing AKI and PGF allografts after baseline adjustment.**
(DOCX)

Table S6 **Highly correlated genes (Spearman's rho >0.7 or <−0.7) to miR-182-5p, miR-132-3p, miR-212-3p and miR-149-3p out of the baseline adjusted differentially regulated gene list.**
(DOCX)
