# Supplementary material for: Molecular Pathogenesis of Post-Transplant Acute Kidney Injury: Assessment of Whole-Genome mRNA and MiRNA Profiles
Source: PLoS One. 2014 Aug 5;9(8):e104164. doi: 10.1371/journal.pone.0104164 (PMC4122455; doi:10.1371/journal.pone.0104164)
Supplement: Figure S1 — Principal component analysis based on the 245 differentially regulated genes comparing post-TX AKI and PGF allografts. The first three principal components (PC) were plotted, capturing 85% of the variance in the dataset. Acute kidney injury allografts (grey spheres) and allografts with primary kidney function (black spheres) form two distinct clusters. (DOCX) [file pone.0104164.s001.docx]

**
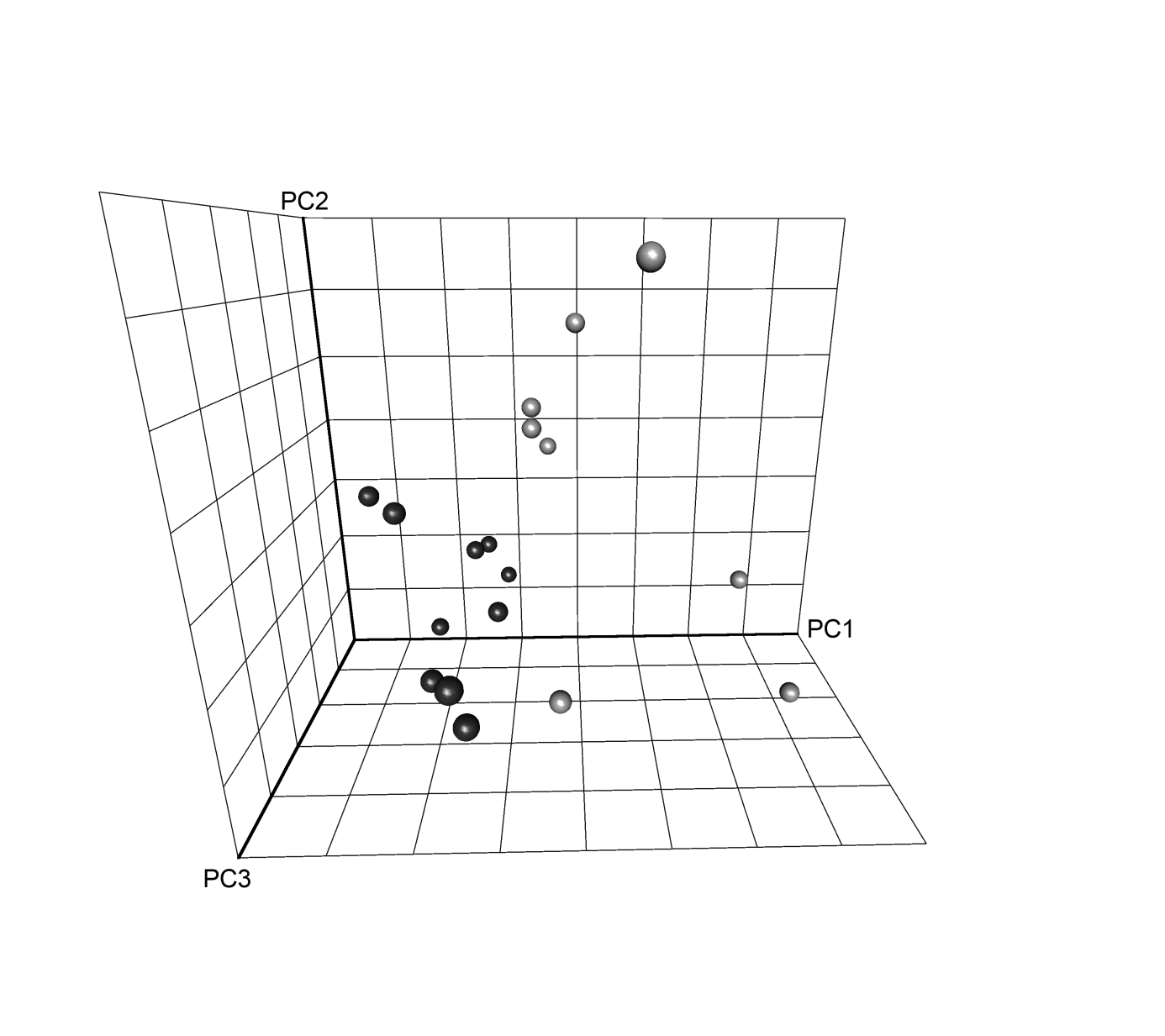
**

# Figure S1. Principal component analysis based on the 245 differentially regulated genes comparing post-TX AKI and PGF allografts. The first three principal components (PC) were plotted, capturing 85% of the variance in the dataset. Acute kidney injury allografts (grey spheres) and allografts with primary kidney function (black spheres) form two distinct clusters.
