# Supplementary material for: Molecular Pathogenesis of Post-Transplant Acute Kidney Injury: Assessment of Whole-Genome mRNA and MiRNA Profiles
Source: PLoS One. 2014 Aug 5;9(8):e104164. doi: 10.1371/journal.pone.0104164 (PMC4122455; doi:10.1371/journal.pone.0104164)
Supplement: Figure S2 — Correlation coefficients (Spearman's rho) of (A) miR-132-3p and (B) miR-212-3p (C) miR-149-3p calculated for all mRNAs against the raw p-values of baseline adjusted mRNA levels between the AKI and PGF group. (DOCX) [file pone.0104164.s002.docx]

**

**

# Figure S2. Correlation coefficients (Spearman’s rho) of (A) miR-132-3p and (B) miR-212-3p (C) miR-149-3p calculated for all mRNAs against the raw p-values of baseline adjusted mRNA levels between the AKI and PGF group.
