# Supplementary material for: Molecular Pathogenesis of Post-Transplant Acute Kidney Injury: Assessment of Whole-Genome mRNA and MiRNA Profiles
Source: PLoS One. 2014 Aug 5;9(8):e104164. doi: 10.1371/journal.pone.0104164 (PMC4122455; doi:10.1371/journal.pone.0104164)
Supplement: Figure S3 — qRT-PCR validation of significantly differentially regulated (A) mRNAs (SLPI, MMP7 and LCN2) and (B) miRNAs (miR-182-5p and miR-21-3p) between AKI and control group (PGF). Log2 (relative expression) values are shown for the qRT-PCR and the array experiment. Individual data points as well as median, 1st and 3rd quartile are provided. (DOCX) [file pone.0104164.s003.docx]

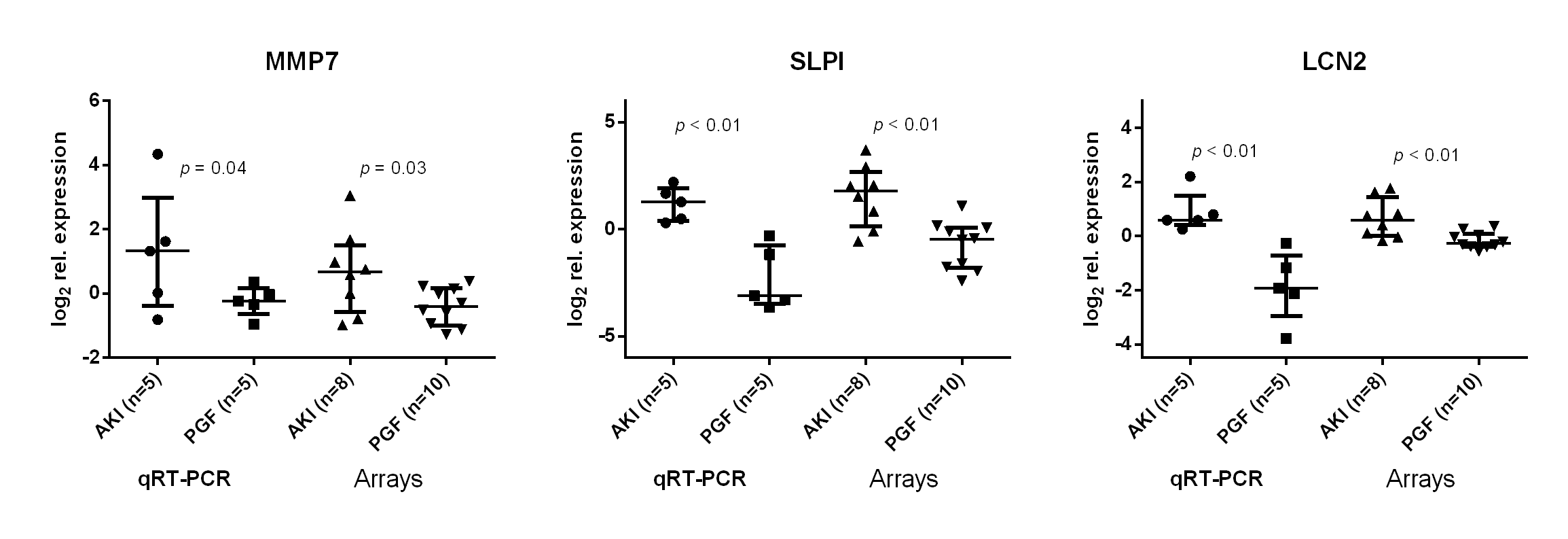

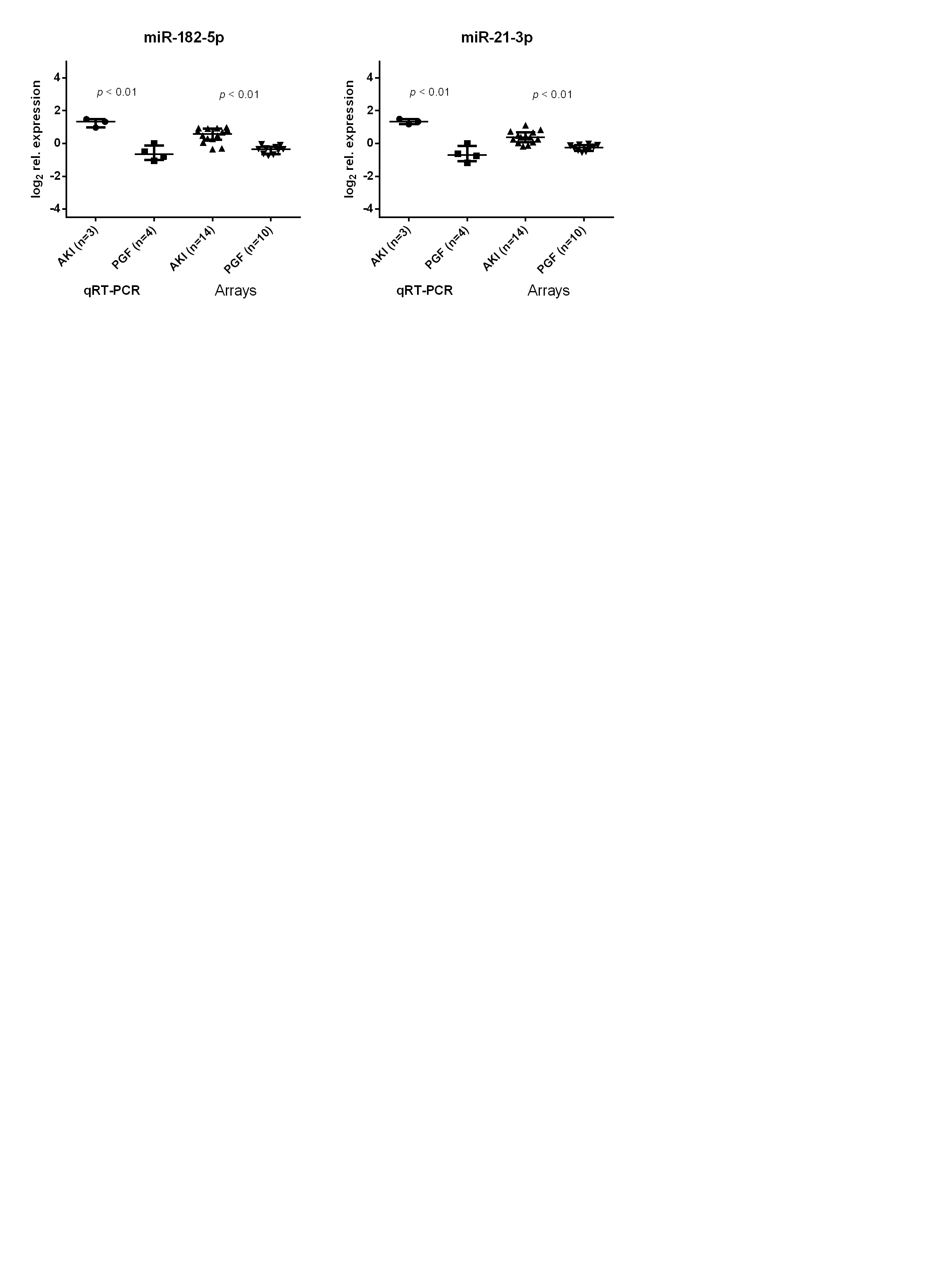


**B**

**A**

# Figure S3. qRT-PCR validation of significantly differentially regulated (A) mRNAs (SLPI, MMP7 and LCN2) and (B) miRNAs (miR-182-5p and miR-21-3p) [[1](#_ENREF_1)] between AKI and control group (PGF). Log_2_ (relative expression) values are shown for the qRT-PCR and the array experiment. Individual data points as well as median, 1^st^ and 3^rd^ quartile are provided.

**qRT-PCR Method**

The TaqMan® MicroRNA Reverse Transcription Kit or High-Capacity cDNA Reverse Transcription Kit with RNase Inhibitor were used to synthesize single stranded cDNA. Real-time PCR was performed using the TaqMan® Gene Expression Master Mix, TaqMan® miRNA expression assays with the ABI 7300 Real-Time PCR System. All instruments and reagents were purchased from Life Technologies. Relative gene expression values were evaluated with the 2^-ΔΔCt^ method using U6 snRNA as control small RNA, PPIA as control mRNA and Stratagene Universal human reference RNA (Stratagene, La Jolla, CA) as reference RNA. qRT-PCR conditions were set according to the manufacturer’s recommendations: 10min 95°C, 40 cycles (15sec 95°C, 1min 60°C) with fluorescence reading during annealing step.

1. Wilflingseder J, Regele H, Perco P, Kainz A, Soleiman A, et al. (2013) miRNA profiling discriminates types of rejection and injury in human renal allografts. Transplantation 95: 835-841.
