# Supplementary material for: Molecular Pathogenesis of Post-Transplant Acute Kidney Injury: Assessment of Whole-Genome mRNA and MiRNA Profiles
Source: PLoS One. 2014 Aug 5;9(8):e104164. doi: 10.1371/journal.pone.0104164 (PMC4122455; doi:10.1371/journal.pone.0104164)
Supplement: Table S1 — 245 significantly differentially regulated mRNAs comparing post-TX AKI and protocol biopsies from allografts with primary graft function. (DOCX) [file pone.0104164.s004.docx]

# Table S1. 245 significantly differentially regulated mRNAs comparing post-TX AKI and protocol biopsies from allografts with primary graft function.

| **Probe Set ID** | **Gene Symbol** | **raw p-value** | **Fold change** | **Gene Description bold…..molecular features discussed as biomarker candidates of AKI** |
| --- | --- | --- | --- | --- |
| 17051827 | AKR1B10 | 9.14E-03 | 7.88 | aldo-keto reductase family 1, member B10 (aldose reductase) |
| 16919547 | SLPI | 1.55E-03 | 5.67 | secretory leukocyte peptidase inhibitor |
| 16971643 | FGB | 3.63E-04 | 5.20 | fibrinogen beta chain |
| 16965606 | SLC34A2 | 4.34E-04 | 3.13 | solute carrier family 34 (sodium phosphate), member 2 |
| 16827679 | NQO1 | 6.00E-03 | 3.01 | NAD(P)H dehydrogenase, quinone 1 |
| 16980836 | FGA | 7.47E-04 | 2.95 | fibrinogen alpha chain |
| **16743647** | **MMP7** | **3.42E-02** | **2.88** | **matrix metallopeptidase 7 (matrilysin, uterine)** |
| 16775083 | OLFM4 | 6.15E-03 | 2.86 | olfactomedin 4 |
| 16924602 | ADAMTS1 | 1.99E-03 | 2.71 | ADAM metallopeptidase with thrombospondin type 1 motif, 1 |
| **17118303** | **COL1A2** | **2.30E-03** | **2.71** | **collagen, type I, alpha 2** |
| 16904193 | ITGB6 | 7.24E-04 | 2.70 | integrin, beta 6 |
| 17000463 | EGR1 | 3.37E-03 | 2.40 | early growth response 1 |
| **16693414** | **S100A8** | **7.43E-04** | **2.40** | **S100 calcium binding protein A8** |
| 17117736 | SOCS3 | 3.71E-03 | 2.36 | suppressor of cytokine signaling 3 |
| 17021437 | CGA | 2.60E-02 | 2.33 | glycoprotein hormones, alpha polypeptide |
| 16997795 | VCAN-AS1 | 1.61E-02 | 2.25 | VCAN antisense RNA 1 (non-protein coding) |
| 16943467 | NFKBIZ | 1.16E-03 | 2.10 | nuclear factor of kappa light polypeptide gene enhancer in B-cells inhibitor, zeta |
| 16981266 | DDX60L | 2.74E-02 | 2.06 | DEAD (Asp-Glu-Ala-Asp) box polypeptide 60-like |
| 16787902 | SERPINA3 | 1.08E-03 | 2.03 | serpin peptidase inhibitor, clade A (alpha-1 antiproteinase, antitrypsin), member 3 |
| **17089525** | **LCN2** | **5.02E-03** | **1.95** | **lipocalin 2** |
| 16760792 | CD163 | 2.77E-02 | 1.94 | CD163 molecule |
| 17000641 | ECSCR | 9.53E-03 | 1.94 | endothelial cell surface expressed chemotaxis and apoptosis regulator |
| 16994434 | DNAH5 | 1.04E-03 | 1.93 | dynein, axonemal, heavy chain 5 |
| 17080648 | HAS2 | 3.27E-02 | 1.91 | hyaluronan synthase 2 |
| 17097661 | TNC | 3.53E-02 | 1.84 | tenascin C |
| 16781606 | ECRP | 2.78E-03 | 1.83 | ribonuclease, RNase A family, 2 (liver, eosinophil-derived neurotoxin) pseudogene |
| 16743874 | CASP5 | 1.81E-02 | 1.83 | caspase 5, apoptosis-related cysteine peptidase |
| 16760928 | CLEC4E | 3.65E-02 | 1.79 | C-type lectin domain family 4, member E |
| 16886491 | TNFAIP6 | 1.71E-02 | 1.79 | tumor necrosis factor, alpha-induced protein 6 |
| 16744572 | NNMT | 4.46E-03 | 1.77 | nicotinamide N-methyltransferase |
| 16986913 | VCAN | 4.35E-02 | 1.77 | versican |
| 16885189 | RNU4ATAC | 1.21E-02 | 1.76 | RNA, U4atac small nuclear (U12-dependent splicing) |
| **17118269** | **NAMPT** | **1.06E-03** | **1.75** | **nicotinamide phosphoribosyltransferase** |
| 16793678 | HIF1A-AS2 | 1.35E-03 | 1.74 | HIF1A antisense RNA 2 (non-protein coding) |
| 16962661 | CLDN1 | 2.41E-02 | 1.73 | claudin 1 |
| 16909021 | SERPINE2 | 5.95E-03 | 1.73 | serpin peptidase inhibitor, clade E (nexin, plasminogen activator inhibitor type 1), member 2 |
| 16749423 | ARNTL2 | 4.60E-03 | 1.72 | aryl hydrocarbon receptor nuclear translocator-like 2 |
| 16871546 | TYROBP | 3.65E-02 | 1.70 | TYRO protein tyrosine kinase binding protein |
| 17016512 | HIST1H2AM | 1.17E-02 | 1.70 | histone cluster 1, H2am |
| 16960911 | LXN | 7.72E-03 | 1.69 | latexin |
| 16974534 | PROM1 | 4.79E-02 | 1.69 | prominin 1 |
| 16968213 | ANXA3 | 6.72E-03 | 1.68 | annexin A3 |
| 16984244 | OSMR | 1.80E-03 | 1.66 | oncostatin M receptor |
| **17002128** | **HAVCR1** | **4.23E-02** | **1.66** | **hepatitis A virus cellular receptor 1** |
| 16984689 | ITGA2 | 1.72E-03 | 1.65 | integrin, alpha 2 (CD49B, alpha 2 subunit of VLA-2 receptor) |
| 17119264 | NOP56 | 2.55E-02 | 1.65 | NOP56 ribonucleoprotein homolog (yeast) |
| 16785048 | FLJ22447 | 2.83E-02 | 1.65 | uncharacterized LOC400221 |
| 17118854 | TAF1D | 1.22E-03 | 1.65 | TATA box binding protein (TBP)-associated factor, RNA polymerase I, D, 41kDa |
| 16909828 | COL6A3 | 4.73E-03 | 1.65 | collagen, type VI, alpha 3 |
| 17118720 | GAS5 | 8.11E-03 | 1.65 | growth arrest-specific 5 (non-protein coding) |
| 17118666 | RABGGTB | 7.57E-03 | 1.64 | Rab geranylgeranyltransferase, beta subunit |
| 17118670 | RPL5 | 2.28E-02 | 1.64 | ribosomal protein L5 |
| 16768297 | DUSP6 | 6.42E-05 | 1.63 | dual specificity phosphatase 6 |
| **16971631** | **TLR2** | **1.18E-02** | **1.63** | **toll-like receptor 2** |
| 16850517 | NDC80 | 2.70E-02 | 1.62 | NDC80 kinetochore complex component homolog (S. cerevisiae) |
| 16903537 | NMI | 1.24E-02 | 1.62 | N-myc (and STAT) interactor |
| 17080486 | TNFRSF11B | 3.23E-03 | 1.62 | tumor necrosis factor receptor superfamily, member 11b |
| 16835158 | ITGB3 | 2.44E-02 | 1.62 | integrin, beta 3 (platelet glycoprotein IIIa, antigen CD61) |
| 16801473 | AQP9 | 1.06E-02 | 1.60 | aquaporin 9 |
| 17045198 | ANLN | 1.16E-02 | 1.59 | anillin, actin binding protein |
| 16690511 | TAF13 | 1.17E-02 | 1.58 | TAF13 RNA polymerase II, TATA box binding protein (TBP)-associated factor, 18kDa |
| 16725227 | MS4A14 | 2.40E-02 | 1.58 | membrane-spanning 4-domains, subfamily A, member 14 |
| 16851397 | RBBP8 | 2.19E-04 | 1.56 | retinoblastoma binding protein 8 |
| 16969439 | ARHGEF38 | 9.83E-03 | 1.56 | Rho guanine nucleotide exchange factor (GEF) 38 |
| 17025560 | SDIM1 | 3.21E-02 | 1.55 | stress responsive DNAJB4 interacting membrane protein 1 |
| 16768941 | IKBIP | 4.58E-03 | 1.54 | IKBKB interacting protein |
| 17110322 | EFHC2 | 3.06E-02 | 1.54 | EF-hand domain (C-terminal) containing 2 |
| 16914264 | WFDC2 | 1.13E-02 | 1.53 | WAP four-disulfide core domain 2 |
| 16734755 | RRM1-AS1 | 2.30E-02 | 1.53 | RRM1 antisense RNA 1 (non-protein coding) |
| 16912379 | TPX2 | 1.36E-02 | 1.53 | TPX2, microtubule-associated, homolog (Xenopus laevis) |
| 16735751 | LYVE1 | 7.55E-04 | 1.53 | lymphatic vessel endothelial hyaluronan receptor 1 |
| 16893704 | FAM110C | 7.62E-03 | 1.52 | family with sequence similarity 110, member C |
| 16707503 | EXOC6 | 8.75E-04 | 1.52 | exocyst complex component 6 |
| 16969686 | CCDC109B | 2.59E-02 | 1.51 | coiled-coil domain containing 109B |
| 16878541 | SPDYA | 1.32E-02 | 1.51 | speedy homolog A (Xenopus laevis) |
| 16991859 | HMMR | 3.75E-02 | 1.51 | hyaluronan-mediated motility receptor (RHAMM) |
| 17024144 | IFNGR1 | 4.61E-03 | 1.51 | interferon gamma receptor 1 |
| 16948021 | ECT2 | 1.82E-03 | 1.51 | epithelial cell transforming sequence 2 oncogene |
| 16720077 | IFITM2 | 3.87E-02 | 1.51 | interferon induced transmembrane protein 2 |
| 16943336 | TMEM45A | 2.14E-03 | 1.50 | transmembrane protein 45A |
| 16761997 | PLCZ1 | 9.81E-04 | 0.67 | phospholipase C, zeta 1 |
| 17102607 | TSPAN7 | 1.11E-03 | 0.67 | tetraspanin 7 |
| 16874249 | SLC6A16 | 1.43E-02 | 0.66 | solute carrier family 6, member 16 |
| 16908575 | SLC23A3 | 1.20E-03 | 0.66 | solute carrier family 23 (nucleobase transporters), member 3 |
| 16899043 | NAT8 | 1.61E-02 | 0.66 | N-acetyltransferase 8 (GCN5-related, putative) |
| 17103717 | CLCN5 | 3.61E-03 | 0.66 | chloride channel, voltage-sensitive 5 |
| 16835816 | ACSF2 | 1.52E-02 | 0.66 | acyl-CoA synthetase family member 2 |
| 16899580 | SUCLG1 | 1.84E-02 | 0.66 | succinate-CoA ligase, alpha subunit |
| 16819264 | MT1X | 4.35E-02 | 0.66 | metallothionein 1X |
| 16745366 | THY1 | 4.41E-04 | 0.66 | Thy-1 cell surface antigen |
| 17095056 | PRUNE2 | 1.42E-03 | 0.66 | prune homolog 2 (Drosophila) |
| 16799106 | SLC12A6 | 6.12E-03 | 0.66 | solute carrier family 12 (potassium/chloride transporters), member 6 |
| 16714944 | PBLD | 1.91E-02 | 0.66 | phenazine biosynthesis-like protein domain containing |
| 16883938 | PLGLA | 3.52E-02 | 0.66 | plasminogen-like A |
| 17015140 | BPHL | 4.60E-03 | 0.65 | biphenyl hydrolase-like (serine hydrolase) |
| 16878081 | KHK | 1.59E-02 | 0.65 | ketohexokinase (fructokinase) |
| 16867558 | FUT6 | 8.20E-03 | 0.65 | fucosyltransferase 6 (alpha (1,3) fucosyltransferase) |
| 17108799 | ARSE | 3.63E-03 | 0.65 | arylsulfatase E (chondrodysplasia punctata 1) |
| 16753270 | SLC16A7 | 1.21E-02 | 0.65 | solute carrier family 16, member 7 (monocarboxylic acid transporter 2) |
| 17002176 | FNDC9 | 2.53E-02 | 0.65 | fibronectin type III domain containing 9 |
| 17055390 | DGKB | 8.04E-04 | 0.65 | diacylglycerol kinase, beta 90kDa |
| 16907546 | GCSHP3 | 2.59E-03 | 0.65 | glycine cleavage system protein H (aminomethyl carrier) pseudogene 3 |
| 16688469 | PTGER3 | 1.56E-03 | 0.65 | prostaglandin E receptor 3 (subtype EP3) |
| 17094766 | TRPM3 | 2.15E-03 | 0.65 | transient receptor potential cation channel, subfamily M, member 3 |
| 17003034 | KIAA1191 | 1.90E-02 | 0.65 | KIAA1191 |
| 16995155 | AGXT2 | 4.04E-02 | 0.65 | alanine--glyoxylate aminotransferase 2 |
| 16965179 | C1QTNF7 | 8.67E-04 | 0.65 | C1q and tumor necrosis factor related protein 7 |
| 16757710 | PEBP1 | 2.58E-04 | 0.64 | phosphatidylethanolamine binding protein 1 |
| 16874097 | HSD17B14 | 3.91E-02 | 0.64 | hydroxysteroid (17-beta) dehydrogenase 14 |
| 17006628 | MCCD1 | 4.97E-03 | 0.64 | mitochondrial coiled-coil domain 1 |
| 16939203 | XYLB | 2.09E-03 | 0.64 | xylulokinase homolog (H. influenzae) |
| 16806999 | GOLGA8A | 3.25E-03 | 0.64 | golgin A8 family, member A |
| 17012761 | TCF21 | 1.49E-03 | 0.64 | transcription factor 21 |
| 16714433 | PCDH15 | 6.30E-03 | 0.64 | protocadherin-related 15 |
| 16816563 | ACSM5 | 2.20E-02 | 0.64 | acyl-CoA synthetase medium-chain family member 5 |
| 16732755 | GRAMD1B | 4.18E-03 | 0.64 | GRAM domain containing 1B |
| 16955535 | ACOX2 | 1.21E-02 | 0.64 | acyl-CoA oxidase 2, branched chain |
| 16995177 | PRLR | 2.90E-02 | 0.64 | prolactin receptor |
| 16816373 | ABCC6P1 | 4.75E-04 | 0.64 | ATP-binding cassette, sub-family C, member 6 pseudogene 1 |
| 17004273 | NQO2 | 9.64E-03 | 0.64 | NAD(P)H dehydrogenase, quinone 2 |
| 16928258 | UPB1 | 7.38E-03 | 0.64 | ureidopropionase, beta |
| 17067284 | EPHX2 | 6.05E-03 | 0.64 | epoxide hydrolase 2, cytoplasmic |
| **16716659** | **RBP4** | **2.08E-02** | **0.63** | **retinol binding protein 4, plasma** |
| 16717970 | CYP17A1 | 1.71E-02 | 0.63 | cytochrome P450, family 17, subfamily A, polypeptide 1 |
| 16777198 | CRYL1 | 7.52E-03 | 0.63 | crystallin, lambda 1 |
| 16693082 | SELENBP1 | 4.01E-04 | 0.63 | selenium binding protein 1 |
| 16701630 | OR2T35 | 4.81E-03 | 0.63 | olfactory receptor, family 2, subfamily T, member 35 |
| 16843549 | CCL14-CCL15 | 3.32E-03 | 0.63 | CCL14-CCL15 readthrough |
| 17078626 | SLC10A5 | 7.28E-03 | 0.63 | solute carrier family 10 (sodium/bile acid cotransporter family), member 5 |
| 16906031 | ZNF385B | 8.17E-04 | 0.63 | zinc finger protein 385B |
| 16796247 | SERPINA6 | 1.23E-03 | 0.62 | serpin peptidase inhibitor, clade A (alpha-1 antiproteinase, antitrypsin), member 6 |
| 17014459 | PLG | 3.80E-02 | 0.62 | plasminogen |
| 16712168 | CUBN | 2.21E-02 | 0.62 | cubilin (intrinsic factor-cobalamin receptor) |
| 17077023 | PXDNL | 3.03E-02 | 0.62 | peroxidasin homolog (Drosophila)-like |
| 16722844 | NELL1 | 7.30E-03 | 0.62 | NEL-like 1 (chicken) |
| 17094946 | TRPM6 | 1.89E-02 | 0.62 | transient receptor potential cation channel, subfamily M, member 6 |
| 17047298 | SPDYE5 | 2.78E-02 | 0.62 | speedy homolog E5 (Xenopus laevis) |
| 17052425 | MGAM | 2.29E-02 | 0.62 | maltase-glucoamylase (alpha-glucosidase) |
| 16808665 | GATM | 4.61E-03 | 0.62 | glycine amidinotransferase (L-arginine:glycine amidinotransferase) |
| 16949322 | FETUB | 6.23E-03 | 0.62 | fetuin B |
| 16966621 | CWH43 | 3.41E-03 | 0.62 | cell wall biogenesis 43 C-terminal homolog (S. cerevisiae) |
| 16858555 | ZNF69 | 7.72E-03 | 0.62 | zinc finger protein 69 |
| 16834603 | TMEM106A | 6.37E-03 | 0.61 | transmembrane protein 106A |
| 16725841 | SCGB1D2 | 2.40E-02 | 0.61 | secretoglobin, family 1D, member 2 |
| 16824588 | GP2 | 2.32E-02 | 0.61 | glycoprotein 2 (zymogen granule membrane) |
| 16682016 | AGMAT | 3.63E-02 | 0.61 | agmatine ureohydrolase (agmatinase) |
| 16827655 | TMED6 | 1.46E-02 | 0.61 | transmembrane emp24 protein transport domain containing 6 |
| 16794632 | ALDH6A1 | 6.98E-03 | 0.61 | aldehyde dehydrogenase 6 family, member A1 |
| 16960844 | VEPH1 | 6.71E-04 | 0.61 | ventricular zone expressed PH domain homolog 1 (zebrafish) |
| 17092331 | PTPRD | 5.29E-03 | 0.61 | protein tyrosine phosphatase, receptor type, D |
| 16968488 | WDFY3-AS2 | 4.42E-03 | 0.60 | WDFY3 antisense RNA 2 (non-protein coding) |
| 16739733 | HRASLS5 | 9.14E-04 | 0.60 | HRAS-like suppressor family, member 5 |
| 16989137 | LEAP2 | 1.83E-04 | 0.60 | liver expressed antimicrobial peptide 2 |
| 16842103 | SHMT1 | 7.98E-03 | 0.60 | serine hydroxymethyltransferase 1 (soluble) |
| 16683300 | TCEA3 | 1.83E-03 | 0.60 | transcription elongation factor A (SII), 3 |
| 16981931 | ENPP6 | 1.52E-02 | 0.60 | ectonucleotide pyrophosphatase/phosphodiesterase 6 |
| 16719892 | ECHS1 | 6.81E-03 | 0.60 | enoyl CoA hydratase, short chain, 1, mitochondrial |
| 16822035 | DPEP1 | 1.84E-02 | 0.60 | dipeptidase 1 (renal) |
| 16800785 | SLC12A1 | 2.04E-02 | 0.59 | solute carrier family 12 (sodium/potassium/chloride transporters), member 1 |
| 16855629 | RNF152 | 1.18E-02 | 0.59 | ring finger protein 152 |
| 16670681 | ANXA9 | 1.37E-03 | 0.59 | annexin A9 |
| 17106852 | XPNPEP2 | 3.76E-02 | 0.59 | X-prolyl aminopeptidase (aminopeptidase P) 2, membrane-bound |
| 17106297 | ZCCHC16 | 9.83E-03 | 0.59 | zinc finger, CCHC domain containing 16 |
| 16815735 | ABAT | 1.47E-02 | 0.59 | 4-aminobutyrate aminotransferase |
| 16782016 | TRAJ56 | 3.13E-02 | 0.59 | T cell receptor alpha joining 56 |
| 17000566 | SLC23A1 | 7.02E-03 | 0.59 | solute carrier family 23 (nucleobase transporters), member 1 |
| 16842673 | SPAG5 | 8.52E-04 | 0.58 | sperm associated antigen 5 |
| 17070360 | RALYL | 1.72E-03 | 0.58 | RALY RNA binding protein-like |
| 16787972 | TCL6 | 3.52E-04 | 0.58 | T-cell leukemia/lymphoma 6 (non-protein coding) |
| 17055472 | SOSTDC1 | 1.34E-02 | 0.58 | sclerostin domain containing 1 |
| 16816579 | ACSM2A | 1.25E-02 | 0.58 | acyl-CoA synthetase medium-chain family member 2A |
| 16960698 | MME-AS1 | 4.87E-02 | 0.58 | MME antisense RNA 1 (non-protein coding) |
| 16969591 | HADH | 4.79E-04 | 0.57 | hydroxyacyl-CoA dehydrogenase |
| 16983765 | NPR3 | 1.69E-03 | 0.57 | natriuretic peptide receptor C/guanylate cyclase C (atrionatriuretic peptide receptor C) |
| 16701620 | OR2T10 | 2.53E-02 | 0.57 | olfactory receptor, family 2, subfamily T, member 10 |
| 17101262 | ARSF | 1.46E-03 | 0.57 | arylsulfatase F |
| 16993065 | SLC34A1 | 5.02E-03 | 0.57 | solute carrier family 34 (sodium phosphate), member 1 |
| 16982699 | SLC6A19 | 7.49E-03 | 0.57 | solute carrier family 6 (neutral amino acid transporter), member 19 |
| **16824602** | **UMOD** | **2.27E-02** | **0.56** | **uromodulin** |
| 16949334 | HRG | 1.07E-02 | 0.56 | histidine-rich glycoprotein |
| 16673763 | TOP1P1 | 3.58E-02 | 0.56 | topoisomerase (DNA) I pseudogene 1 |
| 16871239 | PEPD | 1.75E-02 | 0.56 | peptidase D |
| 16673748 | FMO4 | 1.04E-02 | 0.56 | flavin containing monooxygenase 4 |
| 16698573 | PM20D1 | 1.12E-03 | 0.56 | peptidase M20 domain containing 1 |
| 16780189 | GPC5-AS1 | 1.22E-02 | 0.56 | GPC5 antisense RNA 1 (non-protein coding) |
| **17044253** | **GPNMB** | **1.04E-02** | **0.56** | **glycoprotein (transmembrane) nmb** |
| **17074296** | **DEFB1** | **3.78E-02** | **0.56** | **defensin, beta 1** |
| 16695262 | KCNJ10 | 3.92E-04 | 0.55 | potassium inwardly-rectifying channel, subfamily J, member 10 |
| 16991527 | CYFIP2 | 2.00E-03 | 0.55 | cytoplasmic FMR1 interacting protein 2 |
| 16840723 | SAT2 | 4.62E-03 | 0.55 | spermidine/spermine N1-acetyltransferase family member 2 |
| 17057736 | DDC | 1.46E-02 | 0.55 | dopa decarboxylase (aromatic L-amino acid decarboxylase) |
| 16773086 | FGF9 | 4.37E-04 | 0.55 | fibroblast growth factor 9 (glia-activating factor) |
| 16708728 | SFXN2 | 4.05E-03 | 0.55 | sideroflexin 2 |
| 16800707 | SEMA6D | 8.46E-03 | 0.55 | sema domain, transmembrane domain (TM), and cytoplasmic domain, (semaphorin) 6D |
| 17078783 | SLC7A13 | 4.32E-02 | 0.54 | solute carrier family 7 (anionic amino acid transporter), member 13 |
| 16800061 | CAPN3 | 3.50E-03 | 0.54 | calpain 3, (p94) |
| 16869942 | CYP4F2 | 3.58E-02 | 0.54 | cytochrome P450, family 4, subfamily F, polypeptide 2 |
| 17020059 | GSTA2 | 4.83E-02 | 0.54 | glutathione S-transferase alpha 2 |
| 16850069 | DCXR | 2.39E-02 | 0.54 | dicarbonyl/L-xylulose reductase |
| 16728632 | FOLR1 | 2.01E-03 | 0.54 | folate receptor 1 (adult) |
| 16832634 | PIPOX | 1.28E-02 | 0.54 | pipecolic acid oxidase |
| 16780186 | GPC5-AS2 | 4.92E-02 | 0.54 | GPC5 antisense RNA 2 (non-protein coding) |
| 16669347 | HAO2 | 1.90E-02 | 0.54 | hydroxyacid oxidase 2 (long chain) |
| 17072059 | SLC30A8 | 2.29E-02 | 0.53 | solute carrier family 30 (zinc transporter), member 8 |
| 16949348 | KNG1 | 8.16E-03 | 0.53 | kininogen 1 |
| **16684429** | **FABP3** | **6.78E-04** | **0.53** | **fatty acid binding protein 3, muscle and heart (mammary-derived growth inhibitor)** |
| 17013677 | IYD | 2.91E-03 | 0.52 | iodotyrosine deiodinase |
| 17096030 | FBP1 | 7.99E-03 | 0.52 | fructose-1,6-bisphosphatase 1 |
| 16931716 | MIOX | 2.66E-02 | 0.52 | myo-inositol oxygenase |
| 16808563 | SORD | 1.22E-02 | 0.52 | sorbitol dehydrogenase |
| 16970132 | MYOZ2 | 5.66E-03 | 0.52 | myozenin 2 |
| 16729875 | FOLH1B | 1.73E-02 | 0.51 | folate hydrolase 1B |
| 16679811 | OR2T5 | 2.92E-02 | 0.51 | olfactory receptor, family 2, subfamily T, member 5 |
| 16986072 | TMEM174 | 1.50E-02 | 0.51 | transmembrane protein 174 |
| 17087615 | LPPR1 | 5.54E-04 | 0.51 | lipid phosphate phosphatase-related protein type 1 |
| 16701617 | OR2T34 | 2.26E-02 | 0.51 | olfactory receptor, family 2, subfamily T, member 34 |
| 17020520 | LGSN | 2.76E-02 | 0.51 | lengsin, lens protein with glutamine synthetase domain |
| 16899048 | NAT8B | 3.28E-03 | 0.50 | N-acetyltransferase 8B (GCN5-related, putative, gene/pseudogene) |
| 16664897 | DIO1 | 6.87E-03 | 0.50 | deiodinase, iodothyronine, type I |
| 17007950 | PNPLA1 | 1.15E-03 | 0.50 | patatin-like phospholipase domain containing 1 |
| 16737282 | ELF5 | 3.12E-03 | 0.50 | E74-like factor 5 (ets domain transcription factor) |
| **16773919** | **KL** | **2.97E-03** | **0.49** | **klotho** |
| 16673731 | FMO1 | 2.45E-03 | 0.49 | flavin containing monooxygenase 1 |
| 16716403 | SLC16A12 | 4.57E-04 | 0.49 | solute carrier family 16, member 12 (monocarboxylic acid transporter 12) |
| 16835738 | PPP1R9B | 7.62E-05 | 0.48 | protein phosphatase 1, regulatory subunit 9B |
| 16820157 | HSD11B2 | 1.09E-02 | 0.48 | hydroxysteroid (11-beta) dehydrogenase 2 |
| 16676498 | MFSD4 | 8.10E-03 | 0.48 | major facilitator superfamily domain containing 4 |
| 16973085 | F11 | 1.42E-03 | 0.47 | coagulation factor XI |
| 16976561 | UGT2B11 | 1.39E-02 | 0.47 | UDP glucuronosyltransferase 2 family, polypeptide B11 |
| 16855305 | MRO | 2.41E-03 | 0.46 | maestro |
| 16967743 | AFM | 2.75E-02 | 0.46 | afamin |
| 16756578 | DAO | 2.58E-03 | 0.45 | D-amino-acid oxidase |
| 17075553 | STC1 | 1.53E-02 | 0.45 | stanniocalcin 1 |
| 16701626 | OR2T11 | 6.89E-03 | 0.45 | olfactory receptor, family 2, subfamily T, member 11 |
| **16969729** | **EGF** | **3.23E-03** | **0.43** | **epidermal growth factor** |
| 17001879 | SLC36A2 | 2.87E-02 | 0.43 | solute carrier family 36 (proton/amino acid symporter), member 2 |
| 16687583 | FAM151A | 1.41E-02 | 0.43 | family with sequence similarity 151, member A |
| 16704199 | FXYD4 | 3.68E-02 | 0.41 | FXYD domain containing ion transport regulator 4 |
| 17075589 | NEFL | 4.19E-02 | 0.40 | neurofilament, light polypeptide |
| 16785709 | RDH12 | 3.88E-03 | 0.40 | retinol dehydrogenase 12 (all-trans/9-cis/11-cis) |
| 16934643 | PVALB | 2.02E-04 | 0.39 | parvalbumin |
| 17066278 | LPL | 2.44E-03 | 0.37 | lipoprotein lipase |
| 16834525 | G6PC | 1.46E-02 | 0.34 | glucose-6-phosphatase, catalytic subunit |
| 16962671 | TMEM207 | 1.57E-04 | 0.32 | transmembrane protein 207 |
| 16988781 | CTXN3 | 1.38E-04 | 0.32 | cortexin 3 |
