# Supplementary material for: Molecular Pathogenesis of Post-Transplant Acute Kidney Injury: Assessment of Whole-Genome mRNA and MiRNA Profiles
Source: PLoS One. 2014 Aug 5;9(8):e104164. doi: 10.1371/journal.pone.0104164 (PMC4122455; doi:10.1371/journal.pone.0104164)
Supplement: Table S2 — Significantly over-represented biological processes embedded in the post-TX mRNA signature (245 mRNAs). (DOCX) [file pone.0104164.s005.docx]

# Table S2. Significantly over-represented biological processes embedded in the post-TX mRNA signature (245 mRNAs).

| **Biological process** | **Genes** | **adjusted *p*-value (Benjamini-Hochberg)** |
| --- | --- | --- |
| response to wounding | KNG1, F11, NFKBIZ, NMI, PTGER3, GATM, S100A8, KL, TNC, EPHX2, TLR2, ITGA2, ITGB3, PLG, CD163, TNFAIP6, LYVE1, FGA, FGB, ITGB6, SERPINA3, PEBP1, VCAN, NEFL | 6.82E-04 |
| response to toxin | DDC, CYP17A1, TRPM6, SLC23A1, EPHX2, PEBP1, BPHL, NQO1, NEFL | 9.00E-04 |
| response to metal ion | KHK, TNFRSF11B, AQP9, FGA, GATM, FGB, SLC34A1, ABAT, PEBP1, CAPN3, MT1X | 3.70E-03 |
| oxidation reduction | ACOX2, ALDH6A1, SORD, HSD17B14, NELL1, PIPOX, PXDNL, IYD, FMO4, RDH12, PRUNE2, CYP17A1, FMO1, AKR1B10, MIOX, HAO2, HSD11B2, DAO, CYP4F2, DIO1, NQO1, HADH, DCXR, NQO2 | 3.97E-03 |
| fatty acid metabolic process | ACOX2, LPL, CRYL1, ACSM2A, HAO2, EPHX2, FABP3, ECHS1, CYP4F2, HADH, ACSF2, ACSM5 | 1.77E-02 |
| response to inorganic substance | KHK, TNFRSF11B, AQP9, FGA, GATM, FGB, SLC34A1, ABAT, PEBP1, CAPN3, MT1X, PXDNL | 2.02E-02 |
