# Supplementary material for: Molecular Pathogenesis of Post-Transplant Acute Kidney Injury: Assessment of Whole-Genome mRNA and MiRNA Profiles
Source: PLoS One. 2014 Aug 5;9(8):e104164. doi: 10.1371/journal.pone.0104164 (PMC4122455; doi:10.1371/journal.pone.0104164)
Supplement: Table S3 — 39 significantly differentially regulated mRNAs comparing AKI and PGF allografts after baseline adjustment. (DOCX) [file pone.0104164.s006.docx]

# Table S3. 39 significantly differentially regulated mRNAs comparing AKI and PGF allografts after baseline adjustment.

| **Probe Set ID** | **Gene Symbol** | **raw p-value** | **Fold change** | **Gene Description bold…..molecular features discussed as biomarker candidates of AKI** |
| --- | --- | --- | --- | --- |
| 16919547 | SLPI | 1.20E-03 | 15.17 | secretory leukocyte peptidase inhibitor |
| 17051827 | AKR1B10 | 5.50E-03 | 11.26 | aldo-keto reductase family 1, member B10 (aldose reductase) |
| 16775083 | OLFM4 | 2.98E-03 | 7.88 | olfactomedin 4 |
| 16787902 | SERPINA3 | 2.91E-03 | 7.03 | serpin peptidase inhibitor, clade A (alpha-1 antiproteinase, antitrypsin), member 3 |
| 16827679 | NQO1 | 8.28E-03 | 4.08 | NAD(P)H dehydrogenase, quinone 1 |
| **16743647** | **MMP7** | **1.54E-02** | **4.06** | **matrix metallopeptidase 7 (matrilysin, uterine)** |
| **17118303** | **COL1A2** | **6.93E-03** | **3.40** | **collagen, type I, alpha 2** |
| 16760792 | CD163 | 1.69E-02 | 3.12 | CD163 molecule |
| **16693414** | **S100A8** | **1.66E-02** | **3.09** | **S100 calcium binding protein A8** |
| 17021437 | CGA | 1.18E-02 | 2.91 | glycoprotein hormones, alpha polypeptide |
| 16781606 | ECRP | 5.51E-04 | 2.42 | ribonuclease, RNase A family, 2 (liver, eosinophil-derived neurotoxin) pseudogene |
| 17000641 | ECSCR | 1.34E-02 | 2.40 | endothelial cell surface expressed chemotaxis and apoptosis regulator |
| 16735751 | LYVE1 | 2.09E-03 | 2.28 | lymphatic vessel endothelial hyaluronan receptor 1 |
| **17089525** | **LCN2** | **8.81E-03** | **2.28** | **lipocalin 2** |
| 16760928 | CLEC4E | 1.15E-02 | 1.95 | C-type lectin domain family 4, member E |
| 16948021 | ECT2 | 9.37E-03 | 1.93 | epithelial cell transforming sequence 2 oncogene |
| 16969439 | ARHGEF38 | 5.56E-03 | 1.90 | Rho guanine nucleotide exchange factor (GEF) 38 |
| 17110322 | EFHC2 | 6.80E-03 | 1.88 | EF-hand domain (C-terminal) containing 2 |
| 16994434 | DNAH5 | 5.58E-04 | 1.81 | dynein, axonemal, heavy chain 5 |
| 17118666 | RABGGTB | 1.21E-02 | 1.79 | Rab geranylgeranyltransferase, beta subunit |
| 16707503 | EXOC6 | 1.88E-03 | 1.78 | exocyst complex component 6 |
| 17024144 | IFNGR1 | 7.64E-03 | 1.77 | interferon gamma receptor 1 |
| 16851397 | RBBP8 | 1.30E-03 | 1.76 | retinoblastoma binding protein 8 |
| 16984689 | ITGA2 | 8.27E-04 | 1.74 | integrin, alpha 2 (CD49B, alpha 2 subunit of VLA-2 receptor) |
| 16943336 | TMEM45A | 2.43E-03 | 1.71 | transmembrane protein 45A |
| 16909828 | COL6A3 | 1.03E-02 | 1.70 | collagen, type VI, alpha 3 |
| 16745366 | THY1 | 1.19E-03 | 0.57 | Thy-1 cell surface antigen |
| 17087615 | LPPR1 | 3.00E-03 | 0.57 | lipid phosphate phosphatase-related protein type 1 |
| 16991527 | CYFIP2 | 2.80E-03 | 0.52 | cytoplasmic FMR1 interacting protein 2 |
| 16695262 | KCNJ10 | 3.00E-04 | 0.52 | potassium inwardly-rectifying channel, subfamily J, member 10 |
| 17101262 | ARSF | 3.30E-03 | 0.47 | arylsulfatase F |
| 17094946 | TRPM6 | 2.19E-03 | 0.45 | transient receptor potential cation channel, subfamily M, member 6 |
| 17072059 | SLC30A8 | 4.78E-03 | 0.45 | solute carrier family 30 (zinc transporter), member 8 |
| 16773086 | FGF9 | 8.50E-04 | 0.45 | fibroblast growth factor 9 (glia-activating factor) |
| 16835738 | PPP1R9B | 3.32E-03 | 0.43 | protein phosphatase 1, regulatory subunit 9B |
| 16934643 | PVALB | 2.75E-03 | 0.42 | parvalbumin |
| **16773919** | **KL** | **4.92E-03** | **0.42** | **klotho** |
| 17007950 | PNPLA1 | 1.10E-03 | 0.36 | patatin-like phospholipase domain containing 1 |
| 16962671 | TMEM207 | 9.31E-04 | 0.33 | transmembrane protein 207 |
