# Supplementary material for: Molecular Pathogenesis of Post-Transplant Acute Kidney Injury: Assessment of Whole-Genome mRNA and MiRNA Profiles
Source: PLoS One. 2014 Aug 5;9(8):e104164. doi: 10.1371/journal.pone.0104164 (PMC4122455; doi:10.1371/journal.pone.0104164)
Supplement: Table S4 — Significantly differentially regulated miRNAs comparing post-TX AKI and protocol biopsies from allografts with primary graft function. (DOCX) [file pone.0104164.s007.docx]

# Table S4. Significantly differentially regulated miRNAs comparing post-TX AKI and protocol biopsies from allografts with primary graft function.

| **Probe Set ID** | **miRNA Name** | **miRBase Accession** | **raw p-value** | **Fold change** |
| --- | --- | --- | --- | --- |
| hsa-miR-21-star_st | hsa-miR-21-3p | MIMAT0004494 | 1.64E-02 | 2.22 |
| hsa-miR-3687_st | hsa-miR-3687 | MIMAT0018115 | 1.47E-02 | 1.93 |
| hsa-miR-212_st | hsa-miR-212-3p | MIMAT0000269 | 1.71E-03 | 1.91 |
| hsa-miR-4433_st | hsa-miR-4433-3p | MIMAT0018949 | 6.98E-05 | 1.89 |
| hsa-miR-132_st | hsa-miR-132-3p | MIMAT0000426 | 5.57E-04 | 1.86 |
| hsa-miR-4530_st | hsa-miR-4530 | MIMAT0019069 | 7.70E-03 | 1.85 |
| hsa-miR-1224-5p_st | hsa-miR-1224-5p | MIMAT0005458 | 6.72E-05 | 1.85 |
| hsa-miR-4521_st | hsa-miR-4521 | MIMAT0019058 | 5.49E-04 | 1.84 |
| hsa-miR-885-3p_st | hsa-miR-885-3p | MIMAT0004948 | 2.93E-03 | 1.84 |
| hsa-miR-3648_st | hsa-miR-3648 | MIMAT0018068 | 1.28E-03 | 1.82 |
| hsa-miR-2392_st | hsa-miR-2392 | MIMAT0019043 | 3.22E-03 | 1.76 |
| hsa-miR-4463_st | hsa-miR-4463 | MIMAT0018987 | 1.96E-03 | 1.76 |
| hsa-miR-1587_st | hsa-miR-1587 | MIMAT0019077 | 6.01E-03 | 1.75 |
| hsa-miR-4507_st | hsa-miR-4507 | MIMAT0019044 | 3.08E-03 | 1.73 |
| hsa-miR-193a-3p_st | hsa-miR-193a-3p | MIMAT0000459 | 1.33E-02 | 1.73 |
| hsa-miR-4492_st | hsa-miR-4492 | MIMAT0019027 | 3.12E-02 | 1.71 |
| hsa-miR-27a-star_st | hsa-miR-27a-5p | MIMAT0004501 | 2.29E-02 | 1.69 |
| hsa-miR-1268b_st | hsa-miR-1268b | MIMAT0018925 | 9.24E-04 | 1.68 |
| hsa-miR-4685-5p_st | hsa-miR-4685-5p | MIMAT0019771 | 5.17E-03 | 1.68 |
| hsa-miR-4505_st | hsa-miR-4505 | MIMAT0019041 | 6.64E-03 | 1.67 |
| hsa-miR-4430_st | hsa-miR-4430 | MIMAT0018945 | 3.60E-03 | 1.67 |
| hsa-miR-149-star_st | hsa-miR-149-3p | MIMAT0004609 | 9.76E-04 | 1.67 |
| hsa-miR-4299_st | hsa-miR-4299 | MIMAT0016851 | 5.33E-03 | 1.65 |
| hsa-miR-3679-5p_st | hsa-miR-3679-5p | MIMAT0018104 | 7.20E-03 | 1.63 |
| hsa-miR-106b-star_st | hsa-miR-106b-3p | MIMAT0004672 | 4.57E-02 | 1.62 |
| hsa-miR-939_st | hsa-miR-939-5p | MIMAT0004982 | 1.24E-02 | 1.61 |
| hsa-miR-4749-5p_st | hsa-miR-4749-5p | MIMAT0019885 | 3.25E-03 | 1.60 |
| hsa-miR-1228-star_st | hsa-miR-1228-5p | MIMAT0005582 | 9.39E-03 | 1.60 |
| hsa-miR-1268_st | hsa-miR-1268a | MIMAT0005922 | 1.22E-03 | 1.59 |
| hsa-miR-4508_st | hsa-miR-4508 | MIMAT0019045 | 2.25E-02 | 1.59 |
| hsa-miR-4487_st | hsa-miR-4487 | MIMAT0019021 | 1.58E-02 | 1.58 |
| hsa-miR-762_st | hsa-miR-762 | MIMAT0010313 | 3.33E-03 | 1.58 |
| hsa-miR-3162-5p_st | hsa-miR-3162-5p | MIMAT0015036 | 8.22E-03 | 1.57 |
| hsa-miR-4649-5p_st | hsa-miR-4649-5p | MIMAT0019711 | 8.72E-03 | 1.57 |
| hsa-miR-182_st | hsa-miR-182-5p | MIMAT0000259 | 1.62E-02 | 1.56 |
| hsa-miR-31_st | hsa-miR-31-5p | MIMAT0000089 | 1.13E-02 | 1.56 |
| hsa-miR-4763-3p_st | hsa-miR-4763-3p | MIMAT0019913 | 4.63E-03 | 1.55 |
| hsa-miR-4750_st | hsa-miR-4750-5p | MIMAT0019887 | 1.12E-02 | 1.55 |
| hsa-miR-4690-5p_st | hsa-miR-4690-5p | MIMAT0019779 | 1.06E-02 | 1.54 |
| hsa-miR-4516_st | hsa-miR-4516 | MIMAT0019053 | 3.56E-03 | 1.54 |
| hsa-miR-23a-star_st | hsa-miR-23a-5p | MIMAT0004496 | 2.94E-02 | 1.54 |
| hsa-miR-3656_st | hsa-miR-3656 | MIMAT0018076 | 6.50E-03 | 1.53 |
| hsa-miR-92b-star_st | hsa-miR-92b-5p | MIMAT0004792 | 2.30E-02 | 1.52 |
| hsa-miR-4532_st | hsa-miR-4532 | MIMAT0019071 | 1.55E-02 | 1.52 |
| hsa-miR-3937_st | hsa-miR-3937 | MIMAT0018352 | 2.92E-02 | 1.52 |
| hsa-miR-3141_st | hsa-miR-3141 | MIMAT0015010 | 4.82E-03 | 1.51 |
| hsa-miR-4462_st | hsa-miR-4462 | MIMAT0018986 | 7.11E-03 | 1.51 |
| hsa-miR-4667-5p_st | hsa-miR-4667-5p | MIMAT0019743 | 7.21E-03 | 1.51 |
| hsa-miR-3196_st | hsa-miR-3196 | MIMAT0015080 | 8.67E-03 | 1.50 |
