# Supplementary material for: Molecular Pathogenesis of Post-Transplant Acute Kidney Injury: Assessment of Whole-Genome mRNA and MiRNA Profiles
Source: PLoS One. 2014 Aug 5;9(8):e104164. doi: 10.1371/journal.pone.0104164 (PMC4122455; doi:10.1371/journal.pone.0104164)
Supplement: Table S5 — Significantly differentially regulated microRNAs comparing AKI and PGF allografts after baseline adjustment. (DOCX) [file pone.0104164.s008.docx]

# Table S5. Significantly differentially regulated microRNAs comparing AKI and PGF allografts after baseline adjustment.

| **Probe Set ID** | **miRNA Name** | **miRBase Accession** | **raw p-value** | **Fold change** |
| --- | --- | --- | --- | --- |
| hsa-miR-21-star_st | hsa-miR-21-3p | MIMAT0004494 | 2.41E-02 | 3.34 |
| hsa-miR-212_st | hsa-miR-212-3p | MIMAT0000269 | 6.54E-04 | 2.63 |
| hsa-miR-132_st | hsa-miR-132-3p | MIMAT0000426 | 5.77E-04 | 2.27 |
| hsa-miR-4505_st | hsa-miR-4505 | MIMAT0019041 | 4.75E-02 | 1.95 |
| hsa-miR-3679-5p_st | hsa-miR-3679-5p | MIMAT0018104 | 2.67E-02 | 1.94 |
| hsa-miR-4530_st | hsa-miR-4530 | MIMAT0019069 | 7.45E-03 | 1.94 |
| hsa-miR-4299_st | hsa-miR-4299 | MIMAT0016851 | 6.88E-03 | 1.93 |
| hsa-miR-4433_st | hsa-miR-4433-3p | MIMAT0018949 | 2.41E-03 | 1.93 |
| hsa-miR-4507_st | hsa-miR-4507 | MIMAT0019044 | 2.13E-02 | 1.92 |
| hsa-miR-3648_st | hsa-miR-3648 | MIMAT0018068 | 3.73E-03 | 1.92 |
| hsa-miR-182_st | hsa-miR-182-5p | MIMAT0000259 | 2.46E-03 | 1.88 |
| hsa-miR-1587_st | hsa-miR-1587 | MIMAT0019077 | 4.19E-02 | 1.87 |
| hsa-miR-4667-5p_st | hsa-miR-4667-5p | MIMAT0019743 | 2.58E-02 | 1.87 |
| hsa-miR-1224-5p_st | hsa-miR-1224-5p | MIMAT0005458 | 6.90E-03 | 1.84 |
| hsa-miR-4685-5p_st | hsa-miR-4685-5p | MIMAT0019771 | 4.79E-02 | 1.83 |
| hsa-miR-4430_st | hsa-miR-4430 | MIMAT0018945 | 3.10E-02 | 1.81 |
| hsa-miR-4749-5p_st | hsa-miR-4749-5p | MIMAT0019885 | 1.82E-02 | 1.80 |
| hsa-miR-2392_st | hsa-miR-2392 | MIMAT0019043 | 1.10E-02 | 1.79 |
| hsa-miR-4690-5p_st | hsa-miR-4690-5p | MIMAT0019779 | 2.22E-02 | 1.79 |
| hsa-miR-4463_st | hsa-miR-4463 | MIMAT0018987 | 1.72E-02 | 1.77 |
| hsa-miR-762_st | hsa-miR-762 | MIMAT0010313 | 3.32E-02 | 1.77 |
| hsa-miR-4508_st | hsa-miR-4508 | MIMAT0019045 | 4.49E-02 | 1.72 |
| hsa-miR-149-star_st | hsa-miR-149-3p | MIMAT0004609 | 2.62E-02 | 1.69 |
| hsa-miR-3196_st | hsa-miR-3196 | MIMAT0015080 | 4.28E-02 | 1.67 |
| hsa-miR-4516_st | hsa-miR-4516 | MIMAT0019053 | 1.98E-02 | 1.66 |
| hsa-miR-1268b_st | hsa-miR-1268b | MIMAT0018925 | 2.70E-02 | 1.59 |
| hsa-miR-1268_st | hsa-miR-1268a | MIMAT0005922 | 4.72E-02 | 1.59 |
| hsa-miR-3687_st | hsa-miR-3687 | MIMAT0018115 | 3.02E-02 | 1.57 |
| hsa-miR-4532_st | hsa-miR-4532 | MIMAT0019071 | 4.35E-02 | 1.55 |
