# Supplementary material for: Molecular Pathogenesis of Post-Transplant Acute Kidney Injury: Assessment of Whole-Genome mRNA and MiRNA Profiles
Source: PLoS One. 2014 Aug 5;9(8):e104164. doi: 10.1371/journal.pone.0104164 (PMC4122455; doi:10.1371/journal.pone.0104164)
Supplement: Table S6 — Highly correlated genes (Spearman's rho >0.7 or <−0.7) to miR-182-5p, miR-132-3p, miR-212-3p and miR-149-3p out of the baseline adjusted differentially regulated gene list. (DOCX) [file pone.0104164.s009.docx]

# Table S6. Highly correlated genes (Spearman’s rho > 0.7 or < -0.7) to miR-182-5p, miR-132-3p, miR-212-3p and miR-149-3p out of the baseline adjusted differentially regulated gene list.

|  |  |  |  | **Spearman's rho** | | | |  |
| --- | --- | --- | --- | --- | --- | --- | --- | --- |
| **Probe Set ID** | **Gene Symbol** | **raw p-value** | **Fold change** | **hsa-miR-182-5p** | **hsa-miR-132-3p** | **hsa-miR-212-3p** | **hsa-miR-149-3p** | **Gene Description** |
| 16919547 | SLPI | 1.20E-03 | 15.17 | 0.72 | 0.82 | 0.71 |  | secretory leukocyte peptidase inhibitor |
| 16775083 | OLFM4 | 2.98E-03 | 7.88 |  | 0.71 |  |  | olfactomedin 4 |
| 16787902 | SERPINA3 | 2.91E-03 | 7.03 |  | 0.74 |  |  | serpin peptidase inhibitor, clade A (alpha-1 antiproteinase, antitrypsin), member 3 |
| 16743647 | MMP7 | 1.54E-02 | 4.06 |  | 0.87 | 0.77 |  | matrix metallopeptidase 7 (matrilysin, uterine) |
| 17118303 | COL1A2 | 6.93E-03 | 3.40 |  | 0.89 | 0.82 |  | collagen, type I, alpha 2 |
| 17000641 | ECSCR | 1.34E-02 | 2.40 |  |  | 0.77 |  | endothelial cell surface expressed chemotaxis and apoptosis regulator |
| 17089525 | LCN2 | 8.81E-03 | 2.28 |  | 0.70 |  | 0.71 | lipocalin 2 |
| 16948021 | ECT2 | 9.37E-03 | 1.93 |  | 0.81 |  |  | epithelial cell transforming sequence 2 oncogene |
| 16969439 | ARHGEF38 | 5.56E-03 | 1.90 | 0.77 | 0.81 | 0.77 |  | Rho guanine nucleotide exchange factor (GEF) 38 |
| 17110322 | EFHC2 | 6.80E-03 | 1.88 | 0.70 |  |  |  | EF-hand domain (C-terminal) containing 2 |
| 17118666 | RABGGTB | 1.21E-02 | 1.79 |  | 0.80 | 0.81 |  | Rab geranylgeranyltransferase, beta subunit |
| 16707503 | EXOC6 | 1.88E-03 | 1.78 | 0.77 | 0.87 | 0.76 |  | exocyst complex component 6 |
| 17024144 | IFNGR1 | 7.64E-03 | 1.77 |  | 0.72 |  |  | interferon gamma receptor 1 |
| 16851397 | RBBP8 | 1.30E-03 | 1.76 |  | 0.72 |  |  | retinoblastoma binding protein 8 |
| 16984689 | ITGA2 | 8.27E-04 | 1.74 | 0.72 | 0.75 |  |  | integrin, alpha 2 (CD49B, alpha 2 subunit of VLA-2 receptor) |
| 16943336 | TMEM45A | 2.43E-03 | 1.71 | 0.78 | 0.90 | 0.81 |  | transmembrane protein 45A |
| 16909828 | COL6A3 | 1.03E-02 | 1.70 |  | 0.78 |  |  | collagen, type VI, alpha 3 |
| 16745366 | THY1 | 1.19E-03 | 0.57 | -0.82 | -0.88 | -0.86 |  | Thy-1 cell surface antigen |
| 17087615 | LPPR1 | 3.00E-03 | 0.57 |  | -0.72 |  | -0.72 | lipid phosphate phosphatase-related protein type 1 |
| 16991527 | CYFIP2 | 2.80E-03 | 0.52 |  |  |  | -0.76 | cytoplasmic FMR1 interacting protein 2 |
| 16695262 | KCNJ10 | 3.00E-04 | 0.52 |  |  |  | -0.78 | potassium inwardly-rectifying channel, subfamily J, member 10 |
| 17094946 | TRPM6 | 2.19E-03 | 0.45 |  | -0.81 | -0.81 |  | transient receptor potential cation channel, subfamily M, member 6 |
| 16773919 | KL | 4.92E-03 | 0.42 | -0.72 | -0.86 | -0.74 |  | klotho |
| 17007950 | PNPLA1 | 1.10E-03 | 0.36 |  | -0.70 |  |  | patatin-like phospholipase domain containing 1 |
| 16962671 | TMEM207 | 9.31E-04 | 0.33 |  |  |  | -0.81 | transmembrane protein 207 |
